# The Beginning and Development of the Theranostic Approach in Nuclear Medicine, as Exemplified by the Radionuclide Pair ^86^Y and ^90^Y

**DOI:** 10.3390/ph10020056

**Published:** 2017-06-20

**Authors:** Frank Rösch, Hans Herzog, Syed M. Qaim

**Affiliations:** 1 Institute of Nuclear Chemistry, Johannes Gutenberg University Mainz, Mainz D-55126, Germany; 2 Institute of Neuroscience and Medicine (INM), INM-4 (Physics of Medical Imaging), Research Center Jülich, Jülich D-52425, Germany; h.herzog@fz-juelich.de; 3 Institute of Neuroscience and Medicine (INM), INM-5 (Nuclear Chemistry), Research Center Jülich, Jülich D-52425, Germany; s.m.qaim@fz-juelich.de

**Keywords:** theranostics, ^86^Y, ^90^Y, dosimetry, positron emission tomography, PET

## Abstract

In the context of radiopharmacy and molecular imaging, the concept of theranostics entails a therapy-accompanying diagnosis with the aim of a patient-specific treatment. Using the adequate diagnostic radiopharmaceutical, the disease and the state of the disease are verified for an individual patient. The other way around, it verifies that the radiopharmaceutical in hand represents a target-specific and selective molecule: the “best one” for that individual patient. Transforming diagnostic imaging into quantitative dosimetric information, the optimum radioactivity (expressed in maximum radiation dose to the target tissue and tolerable dose to healthy organs) of the adequate radiotherapeutical is applied to that individual patient. This theranostic approach in nuclear medicine is traced back to the first use of the radionuclide pair ^86^Y/^90^Y, which allowed a combination of PET and internal radiotherapy. Whereas the β-emitting therapeutic radionuclide ^90^Y (t½ = 2.7 d) had been available for a long time via the ^90^Sr/^90^Y generator system, the β^+^ emitter ^86^Y (t½ = 14.7 h) had to be developed for medical application. A brief outline of the various aspects of radiochemical and nuclear development work (nuclear data, cyclotron irradiation, chemical processing, quality control, etc.) is given. In parallel, the paper discusses the methodology introduced to quantify molecular imaging of ^86^Y-labelled compounds in terms of multiple and long-term PET recordings. It highlights the ultimate goal of radiotheranostics, namely to extract the radiation dose of the analogue ^90^Y-labelled compound in terms of mGy or mSv per MBq ^90^Y injected. Finally, the current and possible future development of theranostic approaches based on different PET and therapy nuclides is discussed.

## 1. Introduction and Historical Background

Radioactivity is unique in the sense that it can be routinely used in nuclear medicine both for diagnosis and therapy [1]. Each application, however, demands a special type of radionuclide, the choice being dependent on its decay properties. The underlying principle in diagnostic nuclear medicine is that the radiation dose to the patient is as low as possible, compatible with the required quality of imaging and the diagnostic advantage in comparison to non-radioactive methods. In internal radionuclide therapy (endoradiotherapy), on the other hand, a localized, well-defined radiation dose needs to be deposited in a malignant or inflammatory tissue to achieve the desired therapeutic effect. Thus, for in vivo diagnostic investigations, radionuclides are required that do not cause much radiation dose and can be efficiently detected from outside of the body. To this end, short-lived γ-ray emitters like ^99m^Tc (t½ = 6.0 h), ^123^I (t½ = 13.2 h), ^201^Tl (t½ = 3.06 d), etc., and positron emitters, like ^11^C (t½ = 20.4 min), ^18^F (t½ = 110 min), ^68^Ga (t½ = 67.6 min), etc., are commonly used. As regards internal radionuclide therapy (endoradiotherapy), in general, radionuclides emitting low-range highly ionizing radiation, i.e., α- or β-particles, conversion and/or Auger electrons, have been of great interest. The major problem in internal radiotherapy, however, has been the quantification of radiation dose caused to various organs, mainly due to uncertainties in the measurement of radioactivity from outside of the body of the patient. Although in the case of a few therapeutic radionuclides, e.g., ^131^I (t½ = 8.02 d) and ^188^Re (t½ = 17.0 h), γ-scanning or SPECT has been used to determine the radioactivity distribution in the body, the methodology lacks precision. The uncertainty in radioactivity distribution is still higher for radionuclides decaying by pure β-emission, e.g., ^32^P (t½ = 14.3 d), ^89^Sr (t½ = 50.5 d) and ^90^Y (t½ = 2.7 d), because imaging is usually done through the use of bremsstrahlung. 

In the early 1990s, thoughts started developing in several laboratories to use an SPECT radionuclide as a surrogate of a therapeutic radionuclide [2], e.g., ^111^In (t½ = 2.8 d), a trivalent metal, as a surrogate of ^90^Y, another trivalent metal. The use of an ^111^In-labelled monoclonal antibody (MAb) as a surrogate to carry out biodistribution and imaging studies to be able to do therapy planning with the analogue ^90^Y-MAb continued for many years and has only recently been abandoned. There has also been discussion about the use of several other metallic radionuclides [3,4]. Yet, none of those approaches provided patient-individual quantitative data on radiation doses.

Within the last few years, the combination of both diagnosis (molecular imaging) and therapy (molecular targeted treatment) using one identical (or similar) molecular targeting vector for the same disease is reflected in the term “theranostics”. It is also referred to as “personalized medicine”, which proposes the customization of healthcare with medical decisions, practices and/or products being tailored to the individual patient. Diagnostic testing employed for selecting appropriate therapies is referred to as “companion diagnostics” or “theranostics”. Customized therapeutic products themselves can fall under personalized medicine, as well. Personalized medicine commonly denotes the use of some kind of technology or discovery, enabling a level of personalization not previously feasible or practical. This includes technologies for producing customized pharmaceutical drug products containing individualized dose levels for one or more drug substances.

**Figure 1:**
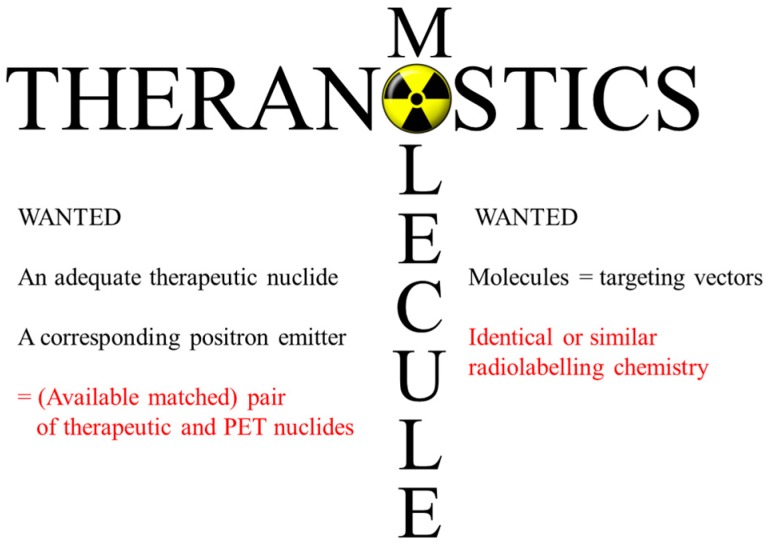
Nuclear and chemical components of radiotheranostics as a prerequisite to extract quantitative radiation doses (i.e., the optimum radioactivity of the radiotherapeutical to inject) for patient-individual treatment based on quantitative (PET) imaging.

In the context of radiopharmacy and molecular imaging, the concept is similar: it is a therapy-accompanying diagnosis with the aim of a patient-specific treatment. Using the adequate diagnostic radiopharmaceutical, the disease and the state of the disease are verified for an individual patient. The other way around, it verifies that the radiopharmaceutical in hand represents a target-specific and selective molecule: the “best one” for that individual patient, cf. Figure 1. Transforming diagnostic imaging into quantitative dosimetric information, the optimum radioactivity (expressed in maximum radiation dose to the target tissue and tolerable dose to healthy organs) of the adequate radiotherapeutical is applied to that individual patient. Finally, post-therapeutic imaging is part of the monitoring of the success of the treatment process for that individual patient.

In terms of radiation dosimetry, the radiodiagnostic version of the targeting vector should reflect the pharmacology of the radiotherapeutic version. Technically speaking, the accuracy of the diagnostic information is best when positron-emitting radionuclides are used within the radiopharmaceutical. Chemically speaking, if the diagnostic version is identical to the therapeutic radiopharmaceutical, the imaging data can be used directly to extract the radiation doses of the therapeutic version. This is commonly denoted by the nomenclature of “matched pairs”, where the diagnostic and therapeutic radionuclides belong to one and the same chemical element. 

In 1992, a few researchers at the Research Center Jülich, Germany, came to the idea of combining PET and endoradiotherapy by using a pair of radionuclides of the same element, one emitting positrons and the other β-particles. This paper intends to illustrate the general approach to radiotheranostics, first exemplified for the pair ^90^Y/^86^Y, which allowed a combination of PET and internal radiotherapy. Whereas the β-emitting therapeutic radionuclide ^90^Y had been available for a long time, the β^+^ emitter ^86^Y (t½ = 14.7 h) had to be developed for medical application. A brief outline of the various aspects of development work (nuclear data, cyclotron irradiation, chemical processing, quality control, etc.) is given. In parallel to the nuclear and radiochemical aspects, the paper discusses the methodology introduced to quantify molecular imaging of ^86^Y-labelled compounds in terms of multiple and long-term PET recordings. Finally, it highlights the ultimate goal of radiotheranostics, namely to extract the radiation dose of the analogue ^90^Y-labelled compound in terms of mGy or mSv per MBq ^90^Y injected in an individual patient. Experience was already available at Jülich in the clinical use of ^90^Y, but the radionuclide ^86^Y needed to be developed for PET studies. In the sections given below, we elaborate the developments in various areas of this systematic work.

## 2. Nuclear Data

### 2.1. Decay Data

The gross features of the decay schemes of the two radionuclides under investigation are known fairly well [5,6,7,8,9,10], though some deficiencies exist in the case of ^86^Y. The main decay data are summarized in Table 1. The therapeutic radionuclide ^90^Y is known to decay 100% by β-emission to stable ^90^Zr (spin: 0^+^), with only one major component having an end point energy of 2290 keV and average β-energy of 933 keV. Only a very small fraction (0.011%) of the β-emission leads to the population of the first excited state of ^90^Zr (spin: 0^+^) at 1761 keV. Since in the monopole transition of ^90^Zr (0^+^ → 0^+^) the emission of a γ-ray is forbidden, the excited state de-excites by internal conversion and pair production, the latter resulting in positron emission of very weak intensity. 

With regard to the diagnostic radionuclide ^86^Y, the decay proceeds via electron capture (EC) and β^+^ emission, followed by emission of a large number of γ-rays. There are six positron groups with varying end-point energies and intensities (see Table 1). The total β^+^ emission intensity amounts to about 33%. This value is rather uncertain, and recommendations have been made to do new measurements utilizing improved methods of detection and quantification [8,9]. Furthermore, a re-evaluation of the whole decay scheme of ^86^Y has also been recommended [10]. On the other hand, it is pointed out that right from the beginning, it was expected that due to rather high positron energy and the presence of several γ-rays in the vicinity of the annihilation radiation, considerable effort would be needed to make use of ^86^Y in PET investigations; see below.

### 2.2. Production Data: Reaction Cross-Sections and Integral Yields

Several routes have been suggested for the production of both ^90^Y and ^86^Y. The former is produced in a nuclear reactor and the latter via charged particle-induced reactions at a cyclotron. Some evaluated data for the two nuclides are available [11]. As regards ^86^Y, the possible methods of production at a small to medium-sized cyclotron include ^86^Sr(p,n)^86^Y, ^86^Sr(d,2n)^86^Y, ^88^Sr(p,3n)^86^Y, ^nat^Rb(^3^He,xn)^86^Y, ^85^Rb(α,3n)^86^Y, ^90^Zr(p,αn)^86^Y and ^nat^Zr(p,x)^86^Y. Some other possible reactions have very low cross-sections.

**Figure 2:**
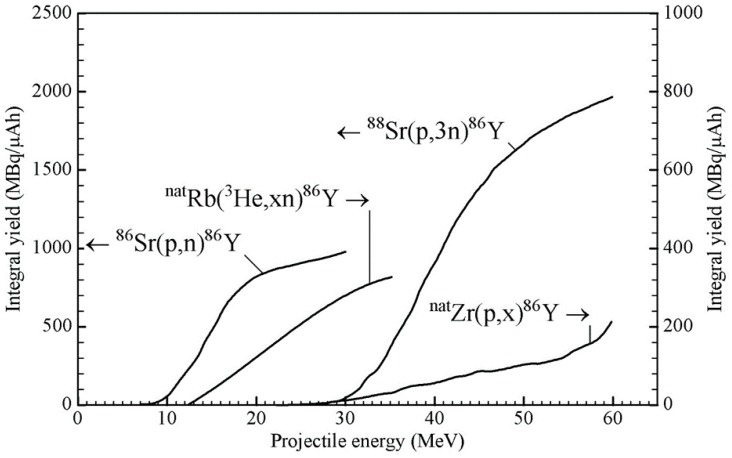
Calculated yields of ^86^Y in ^86^Sr(p,n)^86^Y, ^88^Sr(p,3n)^86^Y, ^nat^Rb(^3^He,xn)^86^Y and ^nat^Zr(p,x)^86^Y reactions (based on experimental and evaluated data given in [15,17,18,19,20,21]), plotted as a function of projectile energy. Note the different yield scales for the first two and the latter two reactions, respectively.

Till 1992, in the literature, some cross-section data existed for several of the above-mentioned reactions [12,13,14]. However, the first systematic study of ^86^Y from the production point of view was performed at the Research Center Jülich [15], and the suitability of the ^86^Sr(p,n)^86^Y reaction was demonstrated [16] using a highly enriched target. Since then, many groups have repeatedly investigated this reaction for production, but no new cross-section measurement has been done. For the ^nat^Rb(^3^He,xn)^86^Y reaction, also cross-sections were measured only once [15], but for the ^nat^Zr(p,x)^86^Y reaction, a few new measurements have been reported over the last few years [17,18,19,20]. Very recently, cross-section data for a few reactions have been evaluated using rigorous nuclear model calculations, and recommended data have been presented [21]. Using those data, the ^86^Y yields from the ^86^Sr(p,n)^86^Y and ^88^Sr(p,3n)^86^Y reactions were calculated, and the results are given in Figure 2 as a function of the projectile energy. To date, those two reactions are the only ones that have been practically used in the production of ^86^Y. As regards other reactions, we consider only the ^nat^Rb(^3^He,xn)^86^Y and ^nat^Zr(p,x)^86^Y processes because they have been fairly well investigated; the expected yields are not very low, and possibilities exist to control the levels of radioactive impurities through the choices of the proper energy ranges in the target. In the case of ^3^He-particle irradiations, the (^3^He,γ) and (^3^He,n) reactions have negligibly small cross-sections [22,23], so that the yields of the impurities ^90^Y, ^88^Y and ^87^Y in the ^nat^Rb(^3^He,xn)^86^Y process at ^3^He-particle energies of < 24 MeV could be expected to be small. Similarly, in the ^nat^Zr(p,x)^86^Y process, the level of the ^87^Y impurity formed via the ^90^Zr(p,α)^87^Y reaction could be minimized if the incident protons have energies >25 MeV where the ^90^Zr(p,αn)^86^Y reaction is favored. We therefore show the integral yields of ^86^Y in Figure 2 also from those reactions, taking the data for the ^nat^Rb(^3^He,xn)^86^Y process from [15] and those for the ^nat^Zr(p,x)^86^Y process from [17,18,19,20,21]. It should be mentioned that in the case of the latter two reactions, the use of highly enriched ^85^Rb and ^9**0**^Zr as targets would increase the ^86^Y yield by about 25 and 100%, respectively, but it would not alter the radioactive impurity level to any significant extent, provided the energy ranges used in the targets are the same as given in Table 2 (see below).

From the available data, the optimum energy ranges for the production of ^86^Y via the four above-mentioned routes were deduced, and the results are given in Table 2. The yield for ^86^Y is the sum of ^86m^Y and ^86g^Y. Considering the associated radioactive impurities, the most preferable route for the production of ^86^Y is the ^86^Sr(p,n)^86^Y reaction on highly enriched ^86^Sr, although the yield of the product is not very high. The small amount of ^87m,g^Y impurity associated with this process was determined in real production experiments and not through calculation. In fact, this impurity should be vanishingly small because the main contributing reaction ^86^Sr(p,γ)^87m,g^Y is expected to have a very small cross-section [24,25]. However, since the enriched ^86^Sr target used had an isotopic enrichment of only 96.4% [16,26,27,28,29,30,31,32,33,34,35,36], the small amount of ^87^Sr (1.33%) present in the target contributed to the formation of the ^87m,g^Y impurity via the ^87^Sr(p,n)-reaction. The same applies to the very small amount of the ^88^Y impurity. It is possibly formed via the ^88^Sr(p,n)^88^Y reaction on ^88^Sr (2.26%) present in the target. Obviously, the levels of both the radionuclidic impurities could be reduced further if the ^86^Sr target used could be of higher enrichment than that given above. An added advantage of the ^86^Sr(p,n)^86^Y route is that a small-sized medical cyclotron is adequate for the production of this radionuclide in quantities sufficient for clinical applications. This reaction has therefore become the method of choice for the production of ^86^Y.

## 3. Production Methodologies

### 3.1. Targetry

**Figure 3:**
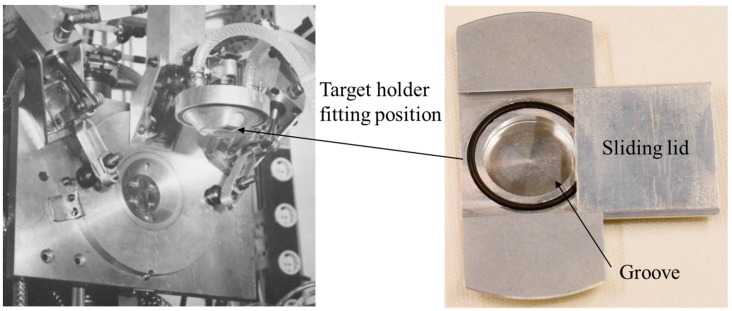
Photograph: An open view of the 4π water-cooled target head at the Forschungszentrum Jülich, used for irradiation (after [37]). The Al target holder with a groove for inserting the ^86^SrCO_3_ pellet is shown on the right. The groove was closed by a sliding lid, and the target holder could be fitted in the target head. After irradiation, the target head was remotely opened, and the target holder fell in a lead pot.

Based on the above discussion, the major efforts to produce ^86^Y have concentrated on the ^86^Sr(p,n)^86^Y reaction, and target systems have been designed for use at low energy cyclotrons (E_p_ ≤ 18 MeV). The originally used highly-enriched ^86^SrCO_3_ as the target material [16] was employed in most of the later works, as well [26,27,28,29,30,31,32,33,34,35,36]. In one case, ^86^SrO was also used [31]. In general, a pressed pellet of the target material was irradiated in a conventional target holder, covered by a thin metal foil and cooled at the back by circulating water. This system could withstand beam currents of up to about 5 μA of 16-MeV protons in irradiations of a few hours in real production runs [16,31,33]. In a modified system [28], the ^86^SrCO_3_ pellet was placed into a groove in a target holder made of Al. The groove was closed by a sliding lid. The target was then fitted in a 4π water-cooled target head, similar to the one described by Michael et al [37]. A photograph of the target system is shown in Figure 3. The primary proton beam energy was 19 MeV, and the energy incident on the target material (after some absorption in the lid) was 16 MeV. The target could withstand beam currents of up to about 10 μA, but during long production runs, nominal currents of about 6 μA were used. In another system [32], besides the water-cooling of the target at the back, chilled He cooling in front of the target was applied, and irradiation currents of up to 15 μA of 11-MeV protons could be used. In yet another system [27,31,35,36], the target material was irradiated with 15-MeV protons at an inclined angle between 6° and 15° in a target holder cooled by a jet of cooling water across its back. A beam current of 10 μA was used [27], but the target may withstand higher currents [31,35,36], at least for short irradiations. Due to limited progress in high-current targetry, the production yield of ^86^Y using a solid target has been so far limited to about 3.5 GBq per batch [28].

In recent years, a new impulse has come to the production of ^86^Y via the ^86^Sr(p,n) reaction at hospital-based cyclotrons. Since those cyclotrons have in general only liquid and gaseous targets (to produce ^18^F, ^11^C, ^15^O, etc.), two conspicuous developments have come up, namely to install solid targetry [38] or to utilize an existing or modified liquid target for irradiating solutions of target isotopes [39,40]. In the latter case, effort is needed to separate radiation-induced chemical species. The yield of ^86^Y achieved using a liquid target is generally low, but it may be enough for local use [40].

Besides the low-energy ^86^Sr(p,n)^86^Y reaction for the production of ^86^Y, the intermediate energy reaction ^88^Sr(p,3n)^86^Y has also been applied in one laboratory [4,41]. A thick ^88^SrCl_2_ pellet was irradiated with protons in the energy range of E_p_ = 45→39 MeV in a well-cooled target holder for 0.5 h at a beam current of 25 μA. The batch yield of ^86^Y achieved was about 4 GBq, with the level of the radionuclidic impurities ^87^Y and ^87m^Y amounting to 5.1% and 56.0%, respectively. The product could thus be used for chemical and labelling studies, but not for clinical investigations.

### 3.2. Chemical Processing

Six major separation methods for ^86^Y from an irradiated strontium target (SrCO_3_, SrO or SrCl_2_) have been suggested. They involve coprecipitation and ion exchange, electrolysis, single column chromatography, multiple column chromatography, solvent extraction and precipitation of the target element. Each method has its own advantages and disadvantages. The method involving coprecipitation and ion exchange was first developed by Rösch et al., [16]. The irradiated enriched ^86^SrCO_3_ was dissolved in a small volume of HCl, and no-carrier-added ^86^Y was coprecipitated with La(OH)_3_ by the addition of NH_4_OH solution. The precipitate was centrifuged off and taken up in a few drops of HCl. The separation of radioyttrium from inactive La was then effected through cation-exchange chromatography by elution with α-hydroxyisobutyric acid, either at normal pressure [16] or, as developed later by Kettern et al., [28], in combination with HPLC. The radioactivity amounting to several GBq was collected in only 150 μL solution. The radionuclidic quality control was done via γ-ray spectrometry, and the chemical purity of the product was checked via inductively-coupled plasma-mass spectrometry (ICP-MS) [28]. In a slight variation of this method [26], the coprecipitation of radioyttrium was done with Fe(OH)_3_, followed by a final purification of ^86^Y by ion-specific resin chromatography.

The electrolytic method of separation was first developed by Reischl et al., [27] and later optimized by Yoo et al., [31] and Lukic et al., [33]. The target was dissolved in dilute HNO_3_ and electrolyzed at 2 A using two Pt plate electrodes. Thereafter, the electrodes were removed from the cell and placed in a second glass vial filled with fresh dilute HNO_3_. A third electrode (platinum wire) was then inserted, and a second electrolysis was performed at 200–400 mA. The ^86^Y activity was collected on the Pt wire from where it was recovered by 250 μL of 0.05 M HCl. 

The single column chromatographic method of separation of radioyttrium was investigated by Kandil et al., [34], using both the cation-exchanger Dowex 50W-X8 (H^−^ form) and the anion-exchanger Dowex 21k (Cl^−^ form). The irradiated ^86^SrCO_3_ was dissolved in 6 M HCl, and the solution was evaporated to near dryness. For cation-exchange separation, the residue was dissolved in 0.1 M citrate buffer, pH 4. For anion-exchange separation, however, the dissolution of the residue was done in 0.1 M citrate buffer, pH 5. The column was loaded with the radioactive solution, and elution was done with 0.1 M citrate buffer, pH 4, while working with the cation-exchange column, or with 0.1 M citrate buffer, pH 5, when using the anion-exchange column. In the former case, ^86^Y passed through the column, while Sr remained on the column. In the latter case, Sr passed through the column, and ^86^Y remained on the column, which was later removed and collected by elution with 1 M HCl. The cation-exchange method was found to be superior because it led to higher chemical purity. However, the final volume of the product was large (~60 mL), and the concentration of the activity was needed. In a similar study, Sadeghi et al., [35,36] investigated the separation of radioyttrium from an irradiated ^nat^SrCO_3_ target via ion-exchange chromatography. The mixture of strontium and radioyttrium was absorbed onto a chelating resin Chelex 100 column (H^+^ form). Strontium and radioyttrium were then eluted sequentially with 0.01 M HNO_3_ and 1 M HNO_3_, respectively. The final volume of the radioyttrium fraction was large (~20 mL). In yet another method [41], the irradiated ^88^SrCl_2_ target was dissolved in 2.5 M HCl and transferred on to an Eichrom DGA resin column. Thereafter, radioyttrium was eluted with 0.5 M HCl in a total volume of about 10 mL. 

The multiple column chromatographic method of the separation of ^86^Y was introduced by Garmestani et al., [29] and further developed by Park et al., [30]. In this method, the irradiated target was dissolved in 4 N HNO_3_, and the solution was loaded onto a Sr-selective resin. On washing the column further with 4 N HNO_3_, radioyttrium was removed and then adsorbed on a second RE-SPEC column. Thereafter, the radioyttrium was further eluted by using 0.1 N HCl and readsorbed on a third Aminex A5 resin column connected in sequence. Finally, ^86^Y was eluted from the Aminex column with 3 N HCl and, after concentration, obtained in 200 μL of 0.1 N HNO_3_ or radiolabeling buffer. Labelling herceptin^TM^ or DOTA-biotin, whereby a specific binding was proven, testified the purity of the product ^86^Y.

The solvent extraction method for the separation of ^86^Y from a ^86^SrCO_3_ target was developed by Kandil et al., [34]. It consisted of dissolution of the irradiated target in 1 M HCl, transfer to a separatory funnel and addition of an equal volume of 10% (*v*/*v*) di-ethylhexyl phosphoric acid (HDEHP) diluted in n-heptane. The mixture was shaken for 3 min. After disengagement, the organic phase was washed with 200 mL of 1 M HCl for 2 min. Then, 9 M HCl led to the recovery of the radioyttrium activity in the aqueous phase. The solution was dried and the residue dissolved in dilute HCl. 

The isolation of radioyttrium from the irradiated ^86^SrCO_3_ by simple precipitation of the target element was developed by Avila-Rodriguez et al., [32]. The target material was dissolved in 0.5 mL of 6 M HCl and made basic by the addition of 5 mL of 1 M NH_4_OH. This solution was loaded onto a chimney supporting a Whatman 42 filter paper and filtered under vacuum. The filter paper was then washed with water to remove traces of strontium. The no-carrier-added ^86^Y was recovered from the filter paper using 1 M HCl. The solution was evaporated to dryness and the activity taken up in 500 μL of 0.1 M HCl. The technique was also applied by Sadeghi et al., [42] after some modifications. From the irradiated ^nat^SrCO_3_ target solution in 8 M HCl, at first, Cu and Zn impurities were removed via anion-exchange chromatography. Thereafter, sulfuric acid was gradually added to the ^86–88^Y/Sr solution under heating, followed by filtration of the SrSO_4_ precipitate. Most of the radioyttrium passed through the filter paper. The solution was evaporated to dryness and the residue taken up in HCl. 

### 3.3. Comparison of Separation Methods

The pertinent data are summarized in Table 3. The solvent extraction method was studied using ^nat^SrCO_3_, and the chemical purity achieved was modest. In all other cases, ^86^SrCO_3_ was used as the target material unless otherwise stated. The method involving coprecipitation of ^86^Y with La(OH)_3_, followed by cation-exchange chromatography led to a pure product in a batch yield sufficient for first clinical application [16]. The method was further optimized, both with respect to targetry and chemical separation [28]; the batch yield increased considerably; and ^86^Y of the highest chemical purity was achieved. The two-step electrolytic process has also been successfully developed, and clinical-scale batch yields have been reported [27,31,33]. Regarding the chemical impurity, only the level of Sr was investigated; it was higher than in the coprecipitation/cation-exchange method. The single column cation-exchange chromatography led to similar results as the electrolytic method. The multiple column chromatography has somewhat lower chemical separation efficiency than the other methods. The chemical impurities were not checked, but the separated ^86^Y was tested by labelling two molecules with satisfactory results. The very simple precipitation and filtration method was found to be effective from the separation and batch yield points of views, but the level of the associated chemical impurity was rather high.

Regarding the radionuclidic purity, it should be emphasized here that all of the chemical separation methods lead approximately to the same result because in each work, the enrichment of the target isotope ^86^Sr used was approximately the same (95.6–97%). The major impurities reported in all works were ^87^Y (~0.4%) and ^87m^Y (<3%). While using ^88^SrCl_2_ as the target material [41] to produce ^86^Y via the ^88^Sr(p,3n)^86^Y reaction, on the other hand, the level of the radionuclidic impurity, as expected, was very high (^87m^Y: 56%). Furthermore, the level of the chemical impurity associated with the single column chromatographic method used was high. 

### 3.4. Concluding Remarks about Production Methodologies

Out of all of the above discussed production routes, the ^86^Sr(p,n)^86^Y reaction over the energy range of E_p_ = 14→7 MeV has become the method of choice because it leads to ^86^Y of the highest radionuclidic purity. The ^87m^Y impurity at the level of <3% can be considerably reduced if ^86^Sr of ~99% enrichment would be used. The target material ^86^SrCO_3_ in the form of a pellet, embedded in a 4π water-cooled Al target holder, withstands 16-MeV proton beams of up to 10 μA. For chemical processing of the irradiated material to obtain ^86^Y, the method involving coprecipitation followed by cation-exchange chromatography appears to lead to the product of the highest chemical purity. So far, the maximum batch yield of about 3.5 GBq was also reported using that method. The two-step electrolytic process is also very useful. 

## 4. ^86^Y for PET Imaging

### 4.1. Radiation Emission Properties of ^86^Y

**Figure 4:**
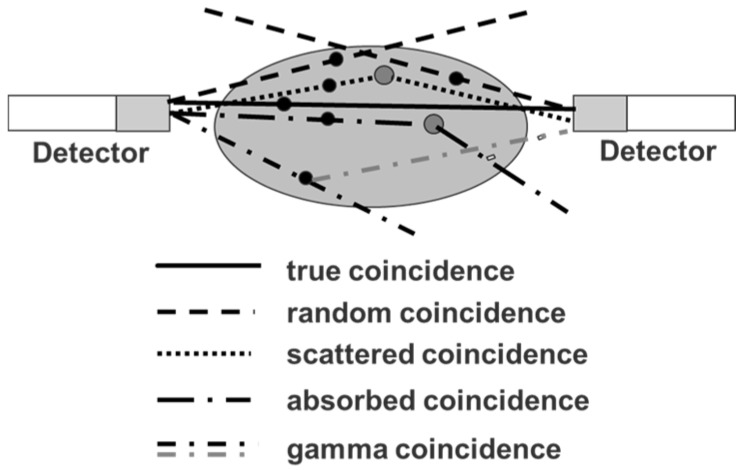
Schematic representation of photon-coincidences. A true coincidence is recorded, if the two annihilation photons from one positron emission hit opposing detectors without interaction. All other coincidences are false and disturb the PET measurement. A gamma coincidence occurs if one annihilation photon from a positron emission and a simultaneously emitted cascade gamma photon hit opposing detectors.

Since the positron abundance of ^86^Y is only 33%, compared to radiotracers labelled with ^18^F (positron abundance of 97%), the PET sensitivity per injected MBq of an ^86^Y-labelled radiotracer is rather low. This is, however, a common feature of many other clinically-adopted positron emitters, as well, such as ^89^Zr (22.3% positron abundance) and ^64^Cu (17.8% positron abundance). Nevertheless, many examples of successful PET-imaging of ^86^Y-labelled radiopharmaceuticals are present in the literature (see Section 5). However, the decay of ^86^Y does not only result in positron emissions with their annihilation photons of 511 keV, but also in the emission of a whole spectrum of additional (single) gamma photons ranging from 443–1921 keV (Table 1). Although many of the gamma photons have energies that are above the upper level of the typical energy window of the PET detector, the detector may accept them after these photons have lost enough energy due to Compton scattering in the subject investigated and/or in the detector crystals. If the single gamma photons, which are accepted by the energy window (ranging, e.g., between 450 and 650 keV), are emitted independent of the positrons, they would just increase the amount of random coincidences and would be corrected together with other random coincidences resulting from those annihilation photons that originate from two different positron emissions, but detected within the coincidence time window. Most of the single gamma photons occurring in the ^86^Y decay are emitted simultaneously with the positron emission (leading to annihilation photons) or electron capture. When one of these photons is accepted by the PET detector together with an annihilation photon within the coincidence time window, this combination is counted as a “coincident PET event”. In the literature, this event is called (cascade) gamma or prompt coincidence. It distorts the “pure” PET recording in addition to other false coincidences, i.e., random and scattered coincidences and to coincidences lost by tissue attenuation and Compton scattering (Figure 4).

The latter three kinds of “non-true” coincidences occur in the same way in the case of the four standard positron emitters ^18^F, ^11^C, ^13^N and ^15^O and can be corrected in a satisfactory way. The gamma coincidences, however, influence the PET measurement qualitatively, as well as quantitatively. Besides ^86^Y, there are other non-standard positron emitters such as ^76^Br or ^124^I that emit cascade gamma photons together with positrons, so that PET measurements are affected by gamma coincidences, as well. On the other hand, different non-standard positron emitters have quite different emission schemes regarding the abundance and energy of the cascade gamma photons. Therefore, a common method for correcting the gamma coincidences has not been achieved. Instead, corrections dedicated to each individual non-standard positron emitter have been suggested as described below for ^86^Y.

### 4.2. Errors Caused by Gamma Coincidences and Their Correction 

**Figure 5:**
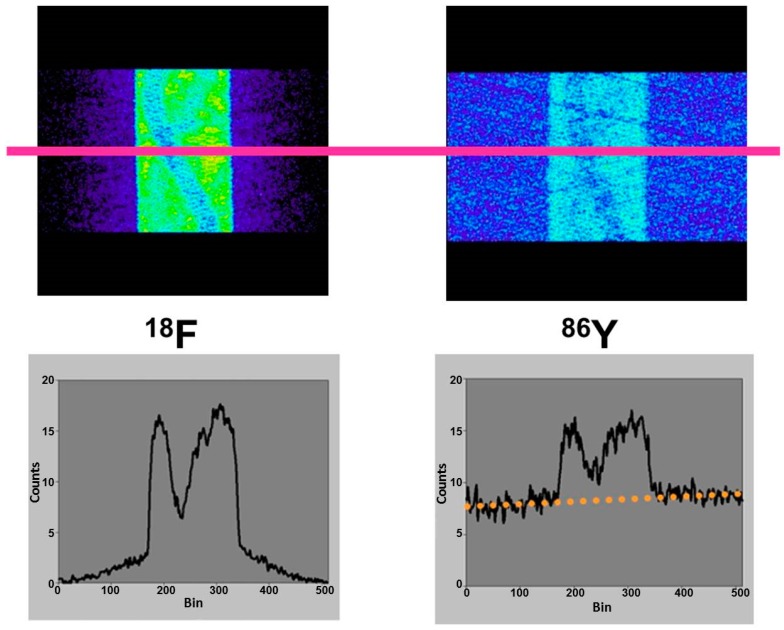
Top row: Sinograms of a rod phantom filled with ^18^F or ^86^Y. Bottom row: Corresponding profiles obtained along the pink line. If the phantom is filled with ^18^F, there are zero counts at the edge of the field of view. If the phantom is filled with ^86^Y, the gamma coincidences lead to a flat background.

Since the directions of the gamma photons and the annihilation photons are not correlated, the gamma coincidences are distributed nearly uniformly within the PET field of view (FOV), causing a primarily flat background in the sinograms, as indicated in Figure 5, in comparison with the equivalent phantom study done with ^18^F. 

**Figure 6:**
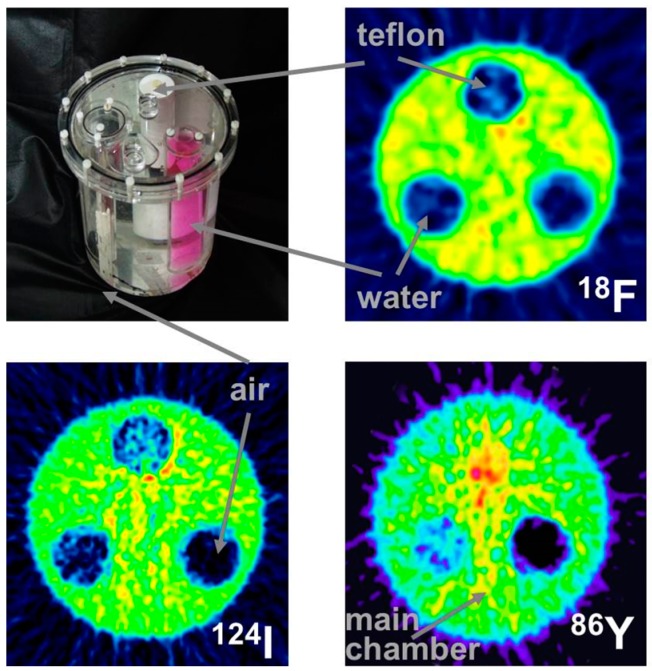
Reconstructed images perpendicular to the central axis of the three-rod phantom, which was filled with ^18^F, ^124^I or ^86^Y (for further details, see the text).

This leads to a corresponding background in reconstructed images and, consequently, to a diminished contrast. Whereas this outcome might be tolerated in the qualitative respect, it leads to quantitative errors, which cannot be accepted. One must consider that the most important motivation for using ^86^Y is the quantitative measurement of its temporal and spatial distribution throughout the body so that the radiation dose caused by the therapeutic ^90^Y can be derived. The quantitative error caused by the gamma coincidences is amplified by the attenuation correction, especially in areas of tissue with a high attenuation coefficient, such as bone. This problem is demonstrated by images of a study with a three-rod phantom (Figure 6). Outside the rods, the phantom was filled with ^18^F, ^124^I or ^86^Y. The three rods were filled with air, (non-radioactive) water and Teflon, which has an attenuation coefficient similar to that of bone. In the image of the phantom filled with ^18^F, all three rod regions look like the background outside the phantom. In contrast, when the phantom was filled with ^86^Y, the water region shows “radioactivity” somewhat less than the radioactivity inside the main chamber. Moreover, in the Teflon region, a considerably erroneous high “radioactivity” is found. In both the water and Teflon regions, the counts caused by gamma coincidences are multiplied by attenuation correction factors, which are especially high in the Teflon (= bone) region. This fact would result in a totally wrong estimation of the radiation dose, in particular in bone metastases, which are among the major aims of ^90^Y-labelled radiotherapeuticals. Figure 6 includes also the findings for ^124^I. The quite different radiation emission scheme of ^124^I results in lower background and correspondingly lower errors compared to ^86^Y. Comparisons between ^124^I and ^86^Y can be found in Herzog et al., [43] and Lubberink and Herzog [44]. 

**Figure 7:**
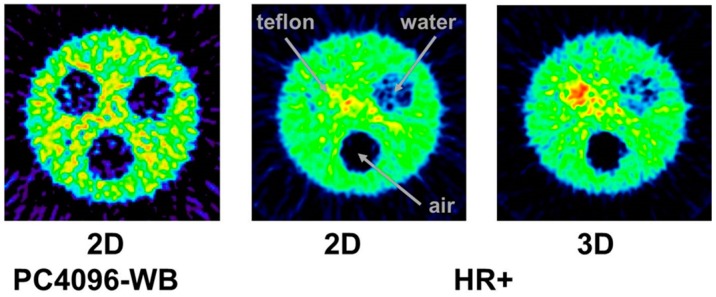
The three-rod phantom filled with ^86^Y was comparatively scanned with the 2D-PET Scanditronix/GE PC4096-WB and with the Siemens/ECAT Exact HR+ operated both in 2D- and 3D-mode (for further details, see the text).

The tests just described were done with the 3D-acquisition mode of the PET detector, i.e., the septa of the PET-Scanner Siemens ECAT Exact HR+ were retracted. When our group performed the first studies with ^86^Y in the early 1990s, all data were acquired with the Scanditronix/GE PET PC4096-WB, in which the detector rings were separated by heavy lead rings, so that the 2D-acquisition was exclusively possible. Compared to 3D-acquisition, considerably less random and scattered coincidences, but also considerably less gamma coincidences, are recorded in 2D-mode. The Scanditronix/GE PC4096-WB had much stronger septa than the Siemens HR+. The septa of the Scanditronix/GE PC4096-WB were 20 cm long and 3 mm thick, whereas those of the Siemens HR+ were 7 cm long and 0.7 cm thick. Considering the construction of the septa of the Scanditronix/GE PC4096-WB, it is not astonishing that an image of the three-rod-phantom recorded with that scanner shows no or only minor (for Teflon) differences between the cold rods and the background outside the phantom (Figure 7). 

The first human study after injection of ^86^Y-citrate in a patient with a history of breast tumor and multiple bone metastases was performed with the Scanditronix/GE PC4096-WB by Herzog et al., in 1993 [45], Section 5.2. This PET scanner was also applied for further investigations: a comparison between ^86^Y-citrate and ^86^Y-EDTMP in ten patients with prostate cancer in 1996 [46] and a study reporting the uptake kinetics of ^86^Y-DOTA-DPhe^1^-Tyr^3^-octreotide in non-human primates by Rösch et al., 1999 [47]. As a conclusion from the experiences with the phantom studies just described, we are quite confident that the uptake and radiation doses reported in those human and non-human studies were reliable. 

The publications by other groups [48,49,50] are not based on the use of the Scanditronix/GE PC4096-WB, but of different scanners with smaller septa (as described above for the Siemens HR+, for example). In our own study with the Siemens HR+ operated in 2D- and 3D-acquisition modes, images of the three-rod-phantom filled with ^86^Y showed false radioactivity uptake in all cold rods (Figure 7). Compared to the ^86^Y activity in the main chamber outside the rods, the “uptake” in the Teflon rod was higher by 107% and 147% for 2D and 3D, respectively. The corresponding findings for the (cold) water rod were 41% and 56%. For the air rod, negative “radioactivity” of −10% and −21%, respectively, was found. Regarding these data, a simple PET measurement without correction for the gamma coincidences will lead to non-negligible errors of the estimated uptake of ^86^Y-labelled diagnostics and consequently of the calculated radiation dose caused by ^90^Y-labelled therapeutics. Because of the smaller errors associated with the 2D-acquisition mode, most of the publications on ^86^Y reported the use of this mode, together with different suggestions for correcting the gamma coincidences. As mentioned above (and indicated in Figure 5), the gamma coincidences cause a flat background in the sinograms so that the correction can be done here before entering the reconstruction including the common corrections, such as for scatter. Therefore, we suggested a linear interpolation between the tales of the single projection lines of the sinogram (yellow line in Figure 5, bottom right) and subtracted this line from the recorded counts [43,51]. 

Looking at two different Siemens PET scanners, ECAT Exact and the ECAT Exact HR+, the best correction was achieved when 100% of the linearly-interpolated counts were subtracted for the first scanner, but only 75% for the second scanner. These findings did not differ between 2D- and 3D-mode. Using a larger phantom, the IEC whole body phantom [52], Kull et al., [50] found that a parabolic approximation to the background caused by the gamma coincidences resulted in a better correction than a mere linear interpolation. More sophisticated methods to remove the gamma coincidences in the sinograms were suggested by Walrand et al., [53], who applied a patient-dependent method based on sinogram tail fitting using an ^86^Y point spread function library, and by Beattie et al., [54], who applied convolution kernels to the entire two-dimensional sinogram (and not just to projection lines). 

**Figure 8:**
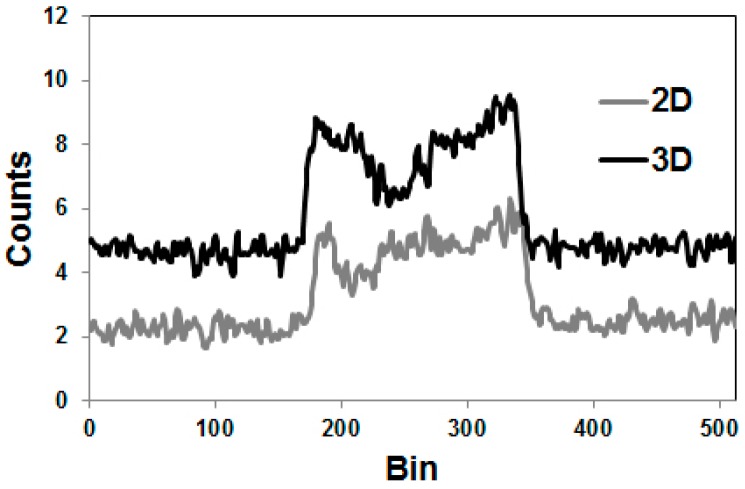
Profiles through sinograms of a rod phantom filled with ^86^Y and recorded with the Siemens/ECAT Exact HR+ operated in both 2D- and 3D-mode. In 3D, the flat background is doubled.

Especially the more sophisticated methods for correcting the gamma coincidences were primarily developed for and tested with the 2D-acquisition, i.e., with septa-in. Nowadays, however, no PET/CT scanner is manufactured with a 2D-option. In 3D-mode, the amount of gamma coincidences is considerably increased (Figure 8). Thus, as indicated above, the reconstructed activity exhibits greater errors if not corrected appropriately (Figure 7). As stated by Walrand et al., [53] and as far as we know, such corrections have not been validated for realistic phantoms. 

A number of studies with ^86^Y-labelled radiopharmaceuticals in small animals have been reported (see Section 6.1). Small animal PET scanners have no septa so that they offer only the 3D-acquisition mode. In spite of that, gamma coincidences play a minor role here. Because of the small size of mice or rabbits, there is much less scattering in the animals, so that most of the high-energy cascade gamma photons do not lose enough energy to be stopped by the detector crystals or to be registered within the energy discrimination window of the detector.

## 5. Radiation Doses of ^90^Y-Therapeuticals Extracted from ^86^Y PET-Imaging

The radionuclide ^90^Y had been intensely used in the 1970s, 1980s and 1990s in the treatment of both benign bone disease [55] and cancer [56,57]. Traditionally, ^90^Y has been considered as a β-emitter with 100% abundance accompanied by very low bremsstrahlung, but without any γ-radiation, cf. International Commission on Radiological Protection (ICRP) Publ. 38, 1983 [58]. It did not seem possible to observe the radioactivity distribution in the patient from outside with an imaging modality such as positron emission tomography (PET), so that radiation doses to tumorous and normal tissue could not be quantitatively derived. 

### 5.1. PET Imaging Based on ^90^Y Positrons 

As mentioned in Section 2.1, a small fraction of the β-decay of ^90^Y ends up in the emission of positrons, generated via the internal pair production. This was reported already in 1955 by Ford [59] and Johnson et al., [60]. Its intensity was accurately measured by Greenberg and Deutsch [61], but a more precise value of 0.003186% was recently published by Selwyn et al., [62]. The first experiments towards PET were, however, performed only in 2004 [63] and showed the general feasibility of this imaging approach. In 2009 and 2010, Lhommel et al., [64] and Werner et al., [65] confirmed the possibility of PET imaging of ^90^Y in the case of selective internal radiotherapy (SIRT) in the liver, where high amounts of radioactivity are locally administered. With radioactivity concentrations in the range of some MBq/mL and acquisition times of half an hour, the low PET sensitivity for ^90^Y is nearly compensated. In a further paper, Lhommel et al., [66] described a specific method to achieve quite accurate estimates of the radiation dose to normal liver and tumor. Recently, Fabbri et al., [67] reported a phantom study towards using ^90^Y for peptide receptor radionuclide therapy. Here, the applied concentration of ^90^Y was also rather high with more than 0.2 MBq/mL. One may be skeptical whether PET-imaging of ^90^Y will be used for further applications. A comprehensive comparison of ^86^Y and ^90^Y can be found in Walrand et al., [53]. 

### 5.2. PET Imaging with ^86^Y Instead of Using ^90^Y

**Figure 9:**
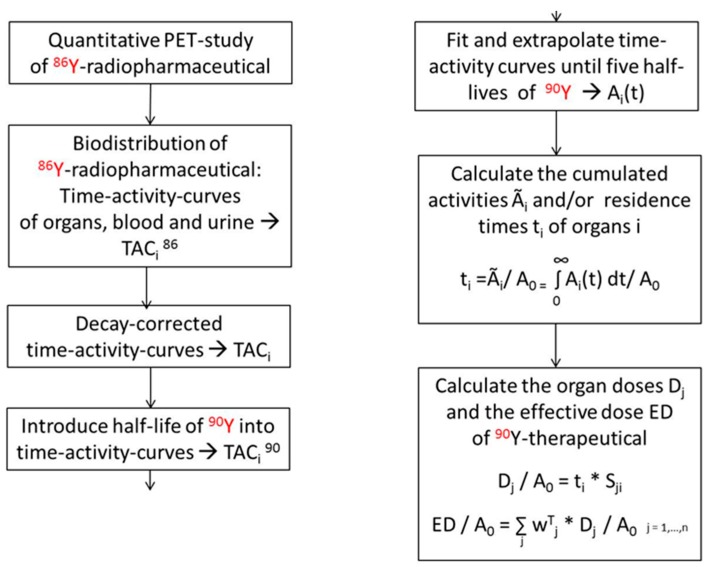
Block-diagram indicating the different steps from the PET study using the ^86^Y-labelled radiopharmaceutical via the extraction of time-activity curves of relevant source organs and tissues to the calculation of the radiation doses D_j_ caused by the ^90^Y-labelled therapeutic to single target organs j and of the effective dose ED, which summarizes the radiation burden for the total body. The factor S_ji_ comprises the mean absorbed dose in a target organ j received per decay in a source organ i. The tissue weighting factors w^T^_j_ are defined by the International Commission on Radiological Protection (ICRP 103) [69]. Commonly, the number of doses is related to the total injected dose A_0_.

If the ^90^Y is substituted by a positron-emitting isotope, the spatial and temporal distribution of yttrium or of a therapeutic compound labelled with yttrium can be measured with PET, especially for applications beyond those where direct PET imaging of ^90^Y is feasible. Among the different isotopes of yttrium, ^86^Y is the most appropriate positron emitter (for its decay properties, cf. Table 1). With respect to uptake, biodistribution and metabolism, ^90^Y and ^86^Y or compounds labelled with ^90^Y and ^86^Y, respectively, do not differ. After the ^86^Y-based biodistribution has been determined by PET quantitatively, the radiation dose caused by ^90^Y can be derived directly using the MIRD formalism [68,69,70]. For this purpose, the S-factors valid for ^90^Y have to be applied instead of those for ^86^Y. The MIRD formalism requires that the measured time-activity curves must be decay-corrected using the half-life of ^86^Y and thereafter multiplied with the decay function of ^90^Y (Figure 9).

### 5.3. The First Human Study with ^86^Y

**Figure 10:**
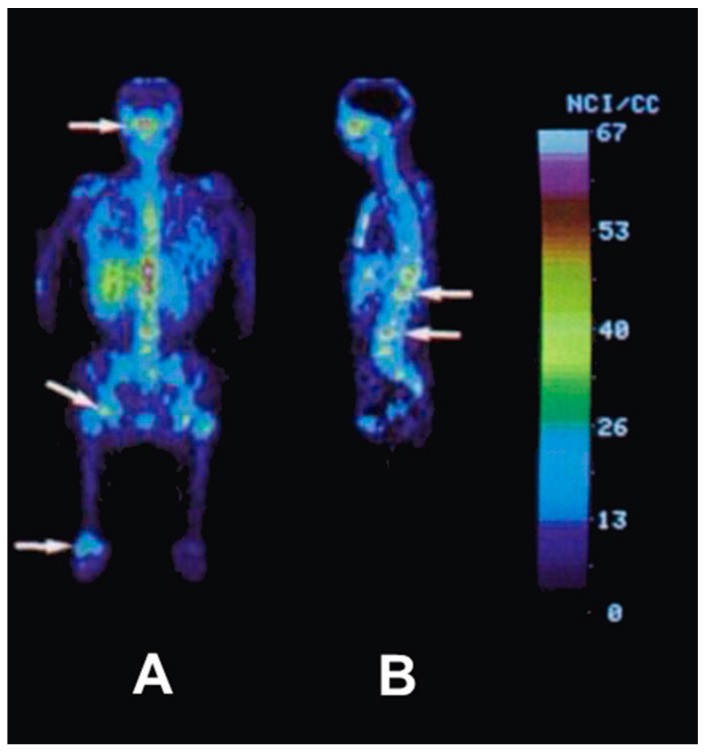
Whole-body images of a female patient with a history of breast cancer 4 h after injection of ^86^Y-citrate. (**A**) Anterior view with coronal slices summed up. (**B**) Eight centimeter-thick sagittal section in the median plane of the patient. The arrows indicate different metastases.

**Figure 11:**
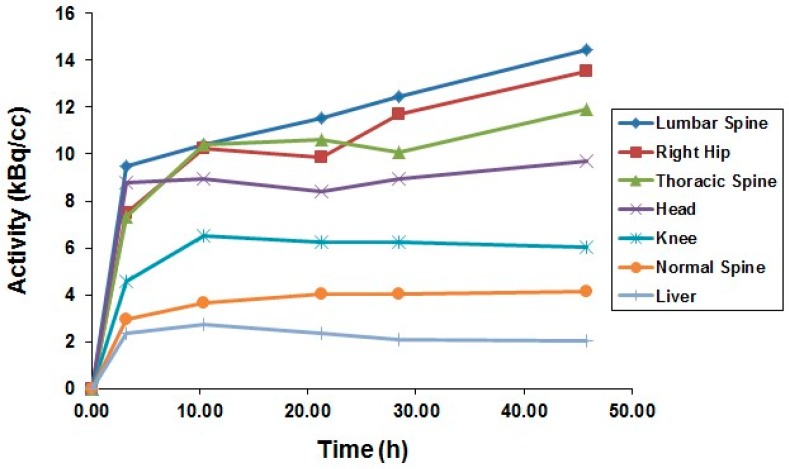
Time-activity curves of ^86^Y-citrate measured in the patient shown in Figure 10. The upper five curves belong to the metastases indicated by arrows in Figure 10.

After the successful development of the production and the radiochemistry of ^86^Y and satisfactory phantom measurements at Jülich, the very first human study applying ^86^Y was performed in a female patient with breast cancer, cf. Herzog et al., in 1993 [45]. In spite of surgical treatment, radiation therapy and chemotherapy, the patient suffered from painful bone lesions and further metastases located in the facial bone, the lung, the pelvis, the right femur and at different locations in the spine. The aim of the PET study, for which the Scanditronix/GE PET PC4096-WB was used, was to deliver data for planning the radiation dose to be delivered by a palliative treatment with ^90^Y-citrate directly after the PET investigation. Following injection of 100 MBq ^86^Y-citrate, whole body scans of 147 cm in length ranging from the head to the most caudally-located metastasis were acquired at 4, 10, 21, 28 and 45 h after tracer injection (p.i.) (Figure 10). The reconstruction with filtered back projection included the common corrections for detector efficiency, dead time, random, scatter and attenuation. Decay-corrected time-activity curves obtained from volumes of interest (VOIs), marking different metastases, normal bone and liver, are displayed in Figure 11. 

The decay-corrected pharmacokinetic data, i.e., percentage of injected ^86^Y activity per organ or per cm^3^ tissue, were considered to be valid also for the therapeutic labelled with ^90^Y. After extrapolating these data to five half-lives of ^90^Y, introducing the decay function of ^90^Y and integrating the extrapolated curves, the cumulated activities Ã were obtained using the MIRD formalism [69], so that the radiation doses caused by 1 MBq of injected ^90^Y to the red marrow, liver and the metastases could be calculated: 1008 μGy, 593 μGy and 3.5 mGy (metastasis with the highest uptake), respectively. 

### 5.4. Comparative Evaluation of ^86^Y-Citrate and ^86^Y-EDTMP 

**Figure 12:**
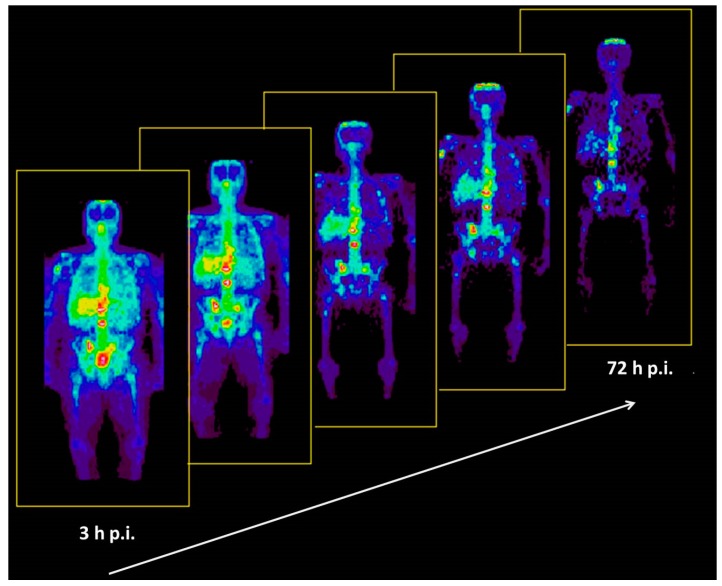
Whole-body distribution of ^86^Y-citrate in a patient from 3–72 h p.i. The anterior view images are summations of all coronal slices.

**Figure 13:**
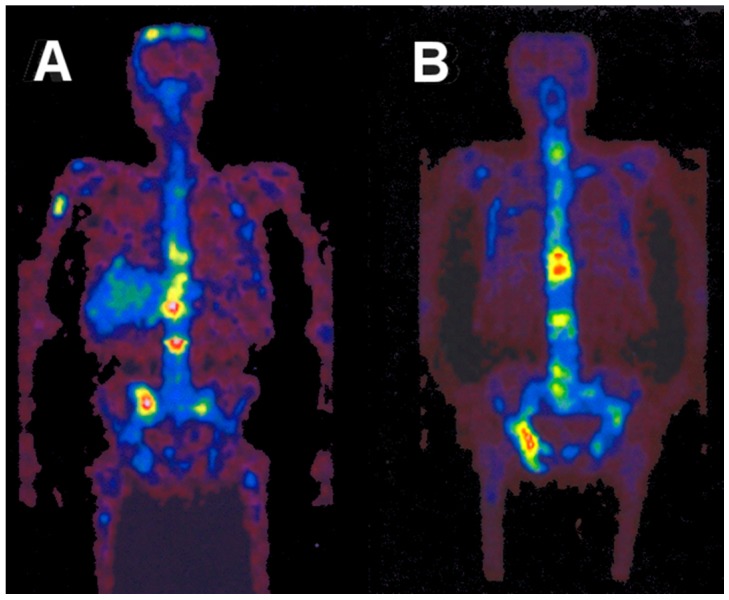
Whole-body images of the distributions of ^86^Y-citrate (**A**) and ^86^Y-EDTMP (**B**) at 22 h p.i. The anterior view images are summations of all coronal slices.

Based on the experience with the above-mentioned investigation, the bio-distributions of ^86^Y-citrate and ^86^Y-EDTMP were compared in ten patients with prostate cancer by Rösch et al., in 1996 [46]. The two radiopharmaceuticals were injected in five patients, each with activities of ^86^Y between 130 and 295 MBq. Using the Scanditronix/GE PET PC4096-WB, a dynamic scan of the upper abdomen was acquired from 0, to 40–90 min p.i. Up to five whole-body PET scans were performed, the first at about 3 h p.i. and the last up to 72 h p.i. (Figure 12). The decisive difference between the two compounds was the lacking liver uptake in the case of ^86^Y-EDTMP (Figure 13). Knowing the ^86^Y-based biodistribution, the radiation doses for ^90^Y-citrate and ^90^Y-EDTMP were estimated for bone metastases, red marrow, liver, kidneys and bladder. The radiation dose to the bone metastases per cm^3^ volume was 26 ± 11 mGy/MBq in the case of ^86^Y-citrate and 18 ± 2 mGy/MBq in the case of ^86^Y-EDTMP. The radiation dose to the red marrow was 2.5 ± 0.4 mGy/MBq and 1.8 ± 0.6 mGy/MBq, respectively. It was concluded that a substantial fraction of ^90^Y-citrate is taken up by the liver and then slowly released from it, so that the concentrations in metastases reached a maximum within about two days and that the corresponding (therapeutic) radiation doses were higher than those with ^90^Y-EDTMP.

### 5.5. Radiation Dose of ^90^Y-DOTA-DPhe^1^-Tyr^3^-Octreotide Based on ^86^Y-DOTA-DPhe^1^-Tyr^3^-Octreotide

**Figure 14:**
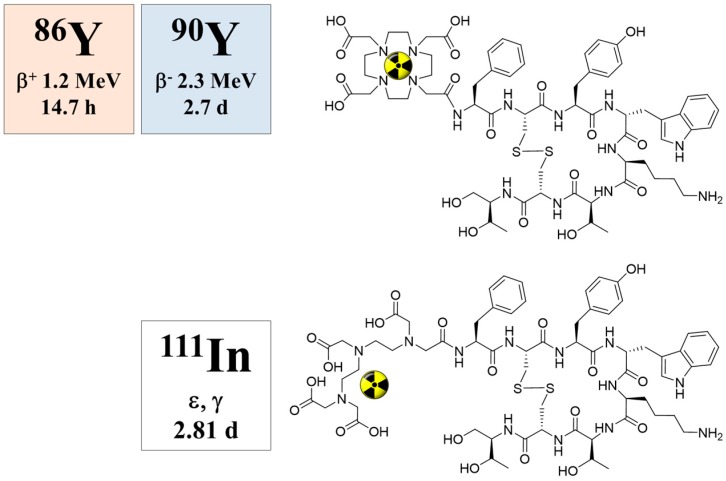
Chemical structures of DOTATOC (for labelling with ^86^Y and ^90^Y) and the DTPA-conjugated octreotide derivative pentreotide (for labelling with ^111^In).

**Figure 15:**
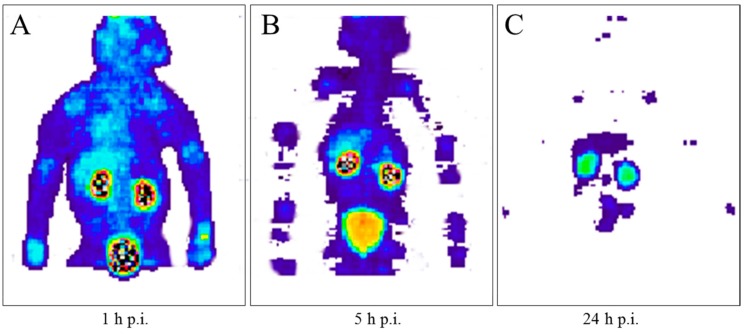
Whole-body PET scans of Baboons A–C injected with 10–40 MBq [^86^Y]DOTA-DPhe^1^-Tyr^3^-octreotide per baboon containing 100 μg/m^2^ peptide recorded at 1 h p.i. (**A**), 5 h p.i. (**B**) and 24 h p.i. (**C**). In this case, there was no simultaneous amino acid co-infusion. The anterior view images are summations of all coronal slices.

When ^90^Y-DOTA-DPhe^1^-Tyr^3^-octreotide (^90^Y-SMT487), cf. Figure 14, was suggested as a promising radiotherapeutic agent for somatostatin receptor-expressing tumors in 1998 (Otte et al., [71]), our group immediately estimated the radiation dosimetry of this compound in non-human primates with the help of the ^86^Y-labelled analogue (Rösch et al., 1999, [47]). Once again, the Scanditronix/GE PET PC4096-WB was applied. Three baboons (two male and one female) aged between three and four years and weighing between 7.9 and 12.4 kg were investigated three times each with intervals of one week. In the first two weeks, the injected ^86^Y-SMT487 contained 100 μg and 2 μg of (cold) SMT487 per m^2^ body surface, respectively. The 100 μg SMT487 reflected the peptide dose in a radiotherapeutic dose of ^90^Y-SMT487, whereas 2 μg SMT487 corresponded to the diagnostic dose of the peptide of <10 μg/m^2^ as used for ^111^In-OctreoScan. In Week 3, an amino acid solution was added to the 100 μg/m^2^ SMT487 protocol. The PET data were acquired over different parts of the baboons from 0–90 min p.i. (first baboon) and at 5 h p.i. (second baboon) and 24 h p.i. (third baboon); Figure 15. For further details, see [47]. Furthermore, radioactivity in blood and urine was measured.

Using the pharmacokinetic data measured with ^86^Y-SMT487, the radiation doses in the case of ^90^Y-SMT487 were derived. The kidneys received the highest radiation dose, with 2.1–3.3 mGy per MBq ^90^Y-SMT487. For the 100 μg/m^2^ SMT487 protocol with amino acid co-infusion, this dose was about 20–40% lower. The liver and the red bone marrow received doses ranging from 0.32–0.53 mGy and 0.03–0.07 mGy per MBq ^90^Y-SMT487, respectively. The average effective dose equivalent amounted to 0.23–0.32 mSv/MBq. Based on those results with comparatively low estimated radiation doses to normal organs, clinical phase 1 trials with ^86^Y-SMT487 in patients with somatostatin receptor-expressing tumors were initiated (see below). 

In 2001, Förster et al., [72] reported “preliminary data on biodistribution and dosimetry for therapy planning of somatostatin receptor positive tumors” using ^86^Y-DOTA-DPhe^1^-Tyr^3^-octreotide (^86^Y-DOTATOC) or ^86^Y-SMT487. Their scanner was a Siemens ECAT Exact acquiring with 2D-mode. Three patients with metastatic carcinoid tumors were examined up to 48 h p.i. with dynamic and static PET scans. Then, 77–186 MBq ^86^Y-DOTATOC were injected. The resulting radiation doses for kidneys, liver and whole body did not differ much from the values deduced from non-human primates [47]; Table 4. In another group of eight patients with progressive metastatic neuroendocrine tumors, the same group estimated the radiation doses comparatively, based on PET measurements with ^86^Y-DOTA-TOC and on combined anterior/posterior gamma scintigraphy with ^111^In-pentetreotide, respectively [73]. Renal protection by administering amino acids was also examined, confirming that the coinjected amino acids lower the radiation dose to the kidneys. While the PET-based radiation doses were in the range of the numbers reported in [47,72], the dose to liver obtained from the ^111^In-pentetreotide scintigraphy was underestimated by 44%, whereas the doses to kidney and the spleen were higher by 16% and 51%, respectively. 

Results of a phase 1 clinical study with ^86^Y-DOTATOC on the pharmacokinetics and biodistribution in 24 patients with somatostatin receptor-positive neuroendocrine tumors and especially at the “renal protective effect of different regimens of amino acid co-infusion” were reported in 2003 [74]. The maximum dose deliverable to the tumor was reached with a mixture of amino acid infused over 10 h. In another paper, that group compared the radiation doses of ^90^Y-DOTATOC to kidneys and tumorous tissues, which were alternatively derived from ^86^Y-DOTATOC PET and ^111^In-DTPA-DPhe^1^-octreotide SPECT [75]. This intra-patient comparison showed that the use of ^86^Y-DOTATOC was more appropriate for the planning of therapy of somatostatin receptor-bearing tumors. Based on the basic research with ^86^Y-DOTATOC just described, the therapeutic ^90^Y-DOTATOC was approved by pharmaceutical administration authorities and introduced to the clinical treatment of neuroendocrine tumors [76,77,78].

## 6. Discussion

### 6.1. Further Use of ^86^Y

As far as we know, no other ^86^Y-labelled peptides or ^86^Y-labelled antibodies have been examined in humans. However, ^86^Y has been used and is being used for a variety of experimental pre-clinical investigations. The main intention is to benefit from the longer half-life of ^86^Y instead of extracting quantitative radiation doses in the context of internal radiation therapy in patients. The focus is on small animal studies. For a review of the many ^86^Y-based PET radiopharmaceuticals: radiochemistry and biological applications, cf. [79,80]; targeted vectors of interest included are tumor receptor binding peptides [81,82,83,84], monoclonal antibodies and antibody fragments [85,86,87,88,89,90] or artificial nanoparticles [91], i.e., constructs with longer retention times. Within this group of investigations, only a few papers yield dosimetric data; however, based on ex vivo organ distribution data in terms of %ID/g, such as Banerjee et al., [84], Cheal et al., [92] and Palm et al., [93].

### 6.2. Alternative Approaches in Radiotheranostics

**Figure 16:**
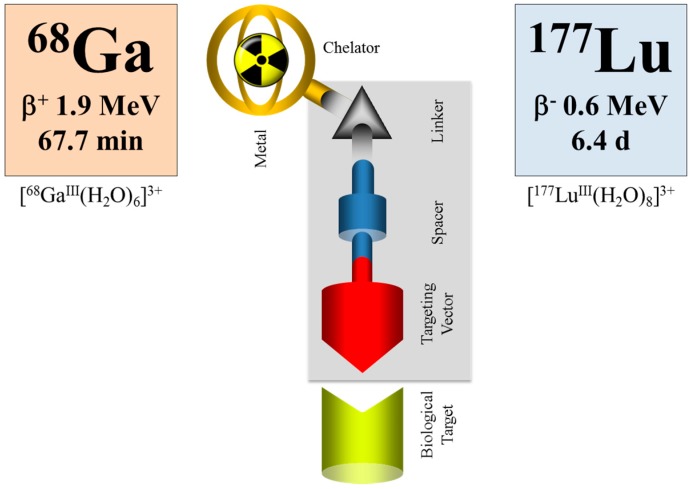
Schematic illustration of the class of tumor targeting vectors (e.g., peptides) conjugated to a chelator, which is able to coordinate a diagnostic and a therapeutic radiometal (e.g., ^68^Ga and ^177^Lu). The typical chelator is DOTA, and for several tracers, there exists a sophisticated linker and spacer chemistry.

Within the last decade, an impressive number of small peptides has been developed, beyond the octreotide class, showing extraordinary promise for the diagnosis and therapy of other than neuroendocrine tumors. Most of them are labelled with the positron emitter ^68^Ga for PET/CT imaging. Some of them are transferred into therapeutic analogues, mainly labelled with ^177^Lu. When applied as the ^68^Ga/^177^Lu pair within the tumor targeting vector (typically DOTA-conjugated), the procedure is referred to as theranostics, as well (e.g., Baum and Rösch [94], Rösch and Baum [95], Velikyan [96]), although a “diagnostic” radionuclide from an element chemically different from the “therapeutic” one is used, cf. Figure 16. 

In such a case, however, pharmacological studies need to ensure that the analogy (rather than identity) in chemistry reflects a similarity in pharmacology for the two differently-radiolabeled compounds. Most probably, this would not be the case, and binding affinities, lipophilicities and pharmacologies will differ, cf., for example, [97,98]. Nevertheless, this deviation between the diagnostic version and the therapeutic version of the radiometal may be handled by introducing a correction factor, similar to the case of ^18^F-FDG (2-deoxy-2-[^18^F]fluoro-D-glucose, a deoxy-glucose derivative) used to quantify glucose consumption rates in units of μmol per mL per minute. ^18^F-FDG does not fully represent the pharmacology of endogenous glucose, but a “lumped constant” introduced in the mathematical interpretation of diagnostic data corrects for that deviation in a way that indeed in vivo FDG data lead to the parameter wanted.

**Figure 17:**
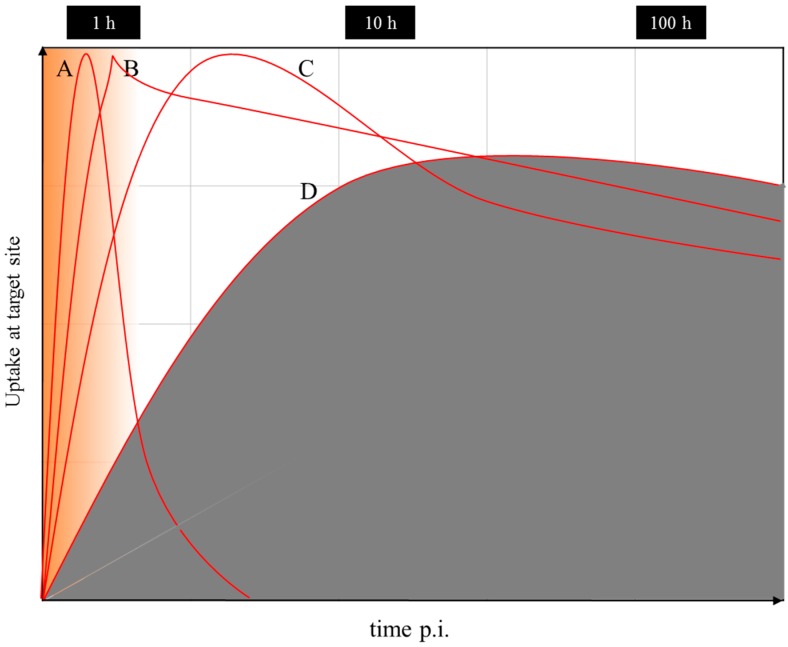
Uptake kinetics of a hypothetical radiotherapeutic compound labelled with, for example, ^90^Y, ^177^Lu or ^225^Ac, in the target tissue is given in grey. Note the logarithmic time scale. (**A**) Example of fast accumulation and fast clearance from the target tissue. (**B**) Example of fast accumulation and slow release. (**C**) Example of medium accumulation and medium release kinetics. (**D**) Example of slow accumulation and slow release. The region of uptake kinetics, which can be covered by a ^68^Ga-labelled analogue, is given in orange.

Nevertheless, even if there is a difference in the chemistry and pharmacology of the two versions of the theranostic molecule, which can be addressed by correction factors, the question remains whether the half-life of the diagnostic radionuclide is able to cover the relevant uptake kinetics of the ^177^Lu-labelled therapeutic as needed for the extraction of dosimetric parameters. Supposing that the pharmacology of a molecule is very similar for the ^68^Ga- (a positron emitting radionuclide of a 67.7-minute half-life for PET) and the ^177^Lu-labelled version (β-emitting radionuclide of a 6.4-day half-life for therapy), ^68^Ga is only able to measure the early uptake kinetics, covering about 4 h post-injection. The “theranostic” measure derived refers to the standard uptake value (SUV) at a certain, early time point only, while it is not possible to obtain radiation doses for the ^177^Lu compound in terms of mGy per MBq injected. The problem is illustrated in Figure 17. The hypothetical uptake kinetics of a radiotherapeutic compound labelled with, for example, ^90^Y, ^177^Lu or ^225^Ac, in the target tissue is given in grey. The region of uptake kinetics, which can be covered by a ^68^Ga-labelled analogue, is given in orange. Obviously, the PET tracer covers only the very early phase of the pharmacology of the analogue compound. It is by far not able to determine the area under the grey curve.

Obviously, the similarity between the physical half-lives of the two radionuclides remains one of the key requirements in radio-theranostics. Currently, ^44^Sc, a positron-emitting radionuclide of a 3.9-h half-life, is attracting interest, as it allows monitoring the uptake kinetics of structurally-similar radiotherapeuticals labelled with, for example, ^177^Lu, cf. [99,100,101]. Indeed, this radionuclide may contribute to the determination of patient-individual radiation dosimetry. However, also in the case of pairs like this with chemically non-identical structures of the two compounds, a combination of ^86^Y/^177^Lu appears to be superior, as the half-life of ^86^Y allows covering more than two days of the pharmacokinetics of the relevant targeting vector. Other promising systems include ^44^Sc (t½ = 3.9 h)/^47^Sc (t½ = 3.35 d), ^64^Cu (t½ = 12.7 h)/^67^Cu (t½ = 2.58 d) and ^83^Sr (t½ = 32.4 h)/^89^Sr (t½ = 50.5 d) [10]. However, many of those radionuclides are not easily available, e.g., ^47^Sc, ^67^Cu, ^83^Sr, etc.

## 7. Conclusions

The concept of theranostics in radiopharmacy and nuclear medicine involves individual patient treatment considering (i) the right therapy (i.e., the adequate targeting vector) applying a potent PET-radiopharmaceutical, (ii) for the right person for a given disease (i.e., the patient expressing the specific target qualitatively in terms of SUV values), (iii) at the right time, (iv) at an individualized radiation dose level (corresponding to the quantitative expression of the specific target) and (v) including the determination of the efficacy of a radiotherapy in terms of post-therapeutic PET imaging.

We believe that our first studies on the radionuclide pair ^86^Y/^90^Y done in 1993 were the starting point of the concept of theranostics. They exemplified the use of radiometal-labelled compounds, i.e., a combination of diagnosis via PET and internal radiotherapy, utilizing two radionuclides of the same element. Both the half-life of ^86^Y and its positron emission property allowed quantification of long-term uptake kinetics and resulted in the ultimate parameter, the radiation dose in terms of mGy or mSv of the ^90^Y analogue per MBq of the injected ^90^Y activity.

In typical present-day examples of so-called theranostics, such as the pair ^68^Ga/^177^Lu, this ultimate goal is not achieved. Although the qualitative aspect, namely the identification of the right therapeutic compound based on the information of an analogue PET tracer for one and the same patient, is now clinical routine for important compounds, yet patients are treated with standard activities of the ^177^Lu-labelled radiotherapeutical, no matter what the quantitative expression of the specific tumor target is. Thus, new approaches are necessary to finally allow for a patient-individual internal ^177^Lu radiation therapy. Radiotheranostics thus still appears to be in a transition phase “between dream and reality”. 

Due to the ease of production of ^86^Y, its 33% abundance of positron emission, its physical half-life of 14.7 h (mimicking the uptake kinetics of most of the long-lived therapeutic radionuclides ^90^Y, ^177^Lu, ^225^Ac, etc.) and due to the chemical similarity of ^86^Y (being a trivalent metal) to nuclides ^90^Y, ^177^Lu, ^225^Ac, etc., we believe that this radionuclide represents a valuable choice for radiotheranostics, provided a sufficient accuracy of quantification is warranted with the 3D PET systems exclusively available today.

## Figures and Tables

**Table 1:** Major decay data of the radionuclide pair ^86^Y and ^90^Y ^(a)^.

Radionuclide	t½	Mode of Decay (%)	E_β(max)_ [keV]	I_β_ [%]	E_γ_ [keV]	I_γ_ [%]
^90^Y	2.7 d	βˉ (100)	2290	100		
^86^Y	14.7 h	EC (67) β^+^ (33)	1043 1248 1603 2019 2335 3153	4.6 16.0 6.2 4.4 1.3 0.6 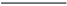 Σ ca. 33	443.1 515.4 580.5 627.8 645.8 703.3 777.6 1076.7 1153.2 1854.3 1920.8	16.9 4.9 4.8 32.6 9.2 15.4 22.4 82.5 30.5 17.2 20.8

^(a)^ Taken from [5,6,7].

**Table 2:** Calculated yields of ^86^Y and relevant isotopic impurities as % of ^86^Y.

Nuclear Reaction	Energy Range	Thick Target Yield of ^86^Y	Radionuclidic Impurities (%)				
	**(MeV)**	**(MBq/μAh)**	**^85^Y** **(t½ = 2.7 h)**	**^85m^Y** **(t½ = 4.9 h)**	**^87g^Y** **(t½ = 3.1 d)**	**^87m^Y** **(t½ = 13.4 h)**	**^88^Y** **(t½ = 106.6 d)**
^86^Sr(p,n)^86^Y ^a^	14→7	371	<0.1	<0.1	0.4 ^d^	1–3 ^d^	0.1 ^d^
^88^Sr(p,3n)^86^Y ^a^	43→33	1005	35.5	2.8	5.4	27.7	0.04
^nat^Rb(^3^He,xn)^86^Y ^b^	24→12	190	71	208	12	46	0.2 ^d^
^nat^Zr(p,x)^86^Y ^c^	40→25	50	unknown	unknown	46	6	0.3

^a^ Values calculated from the evaluated cross-section data [21], unless otherwise stated. ^b^ Values calculated from the experimental data [15]. ^c^ Values calculated from the experimental data and model calculation [17,18,19,20,21]. ^d^ Experimentally-determined values [15,27,28,29,30,31,32,33,34].

**Table 3:** Comparison of chemical separation methods of ^86^Y from the ^86^SrCO_3_ target used in real production runs via the (p,n) reaction (unless otherwise stated).

Method	Typical Irradiation Conditions			Efficiency of Chemical Separation (%)	Typical Production Batch Yield (GBq)	Chemical Impurities (ng/mL)				Ref.
	Energy Range (MeV)	Beam Current (μA)	Time of Irradiation (h)			Fe	Sr	Y	La	
Coprecipitation and ion-exchange	12→8 16→10 15→6	4 6 -	3 4 -	90 90 -	1.4 3.5 1.3	2.3	2.6	<0.02	<0.1	[16] [28] [26]
Electrolysis	15.1→0 14.5→6 14.5→6 14.2→8	10 2 4 5	2.5 4 1 0.5	90 90 90 91	1.2 1.3 ^a^ 0.34 0.36		<100 700			[27] [31] [31] [33]
Single column cation-exchange	16→12 45→39	1 25	3 0.5	91 90	0.45 5.0 ^b^	2 × 10^5^	500 2 × 10^6^			[34] [41]
Multiple column chromatography	13.8→6.1	10	1	80	0.70					[29]
Solvent extraction	16→10	1	3	89	0.48 ^c^		1 × 10^3^			[34]
Precipitation	11→6.1	10	2	88	0.89		1.5 × 10^4^			[32]

^a^ Using the ^86^SrO target. ^b^ Using the ^88^SrCl_2_ target, utilizing the ^88^Sr(p,3n)^86^Y reaction. ^c^ Extrapolated value from the ^nat^SrCO_3_ target.

**Table 4:** Anticipated radiation doses due to the use of ^90^Y-DOTATOC in humans and derived from PET studies with ^86^Y-DOTATOC in baboons and humans.

Organ	Dose per Organ (mGy/MBq)			
	Baboon Study Rösch et al., [47]	Baboon Study Rösch et al., with Amino Acid Cocktail Co-infusion [47]	Human Study Förster et al., [72]	Human Study Helisch et al., [73]
**Intestine wall**	-	-	0.05 ± 0.002	-
**Kidneys**	2.81	2.11	2.73 ± 1.41	1.71 ± 0.89
**Liver**	0.32	0.34	0.660 ± 0.002	0.72 ± 0.04
**Other tissue**	-	-	0.05 ± 0.002	-
**Red marrow**	0.07	0.03	0.05 ± 0.002	0.60 ± 0.02
**Spleen**	-	-	2.32 ± 1.97	2.19 ± 1.11
**Urinary bladder wall**	0.64	0.65	1.03 ± 0.23	-
**Effective dose (mSv/MBq)**	0.25	0.23	0.22 ± 0.07	0.17 ± 0.10

## References

[1] Stöcklin G., Qaim S.M., Rösch F. (1995). The impact of radioactivity on medicine. Radiochim. Acta.

[2] Zimmer A.M., Kuzel T.M., Spies W.G., Duda R.B., Webber D.I., Kazikiewicz J.M., Radosevich J.A., LoCicero J., Robinson P.G., Gilyon K.A. (1992). Comparative pharmacokinetics of In-111 and Y-90 B72.3 in patients following single dose intravenous administration. Antib. Immunoconjug. Radiopharm..

[3] Mausner L.F., Srivastava S.C. (1993). Selection of radionuclides for radioimmunotherapy. Med. Phys..

[4] Srivastava S.C. (2011). Paving the way to personalized medicine: Production of some theragnostic radionuclides at Brookhaven National Laboratory. Radiochim. Acta.

[5] Lederer C.M., Shirley V.S. (1978). Table of Isotopes.

[6] Eckerman K.F., Endo A. (2007). Radionuclide Decay Data and Decay Schemes.

[7] Evaluated Nuclear Structure and Decay File (ENSDF), BNL, USA. www.nndc.bnl.gov/ensdf.

[8] Qaim S.M. (2008). Decay data and production yields of some non-standard positron emitters used in positron emission tomography. Q. J. Nucl. Med. Mol. Imaging.

[9] Qaim S.M. (2011). Development of novel positron emitters for medical applications: Nuclear and radiochemical aspects. Radiochim. Acta.

[10] Qaim S.M. (2017). Nuclear data for production and medical application of radionuclides: Present status and future needs. Nucl. Med. Biol..

[11] Qaim S.M., Tárkányi F., Capote R. (2011). Nuclear Data for the Production of Therapeutic Radionuclides.

[12] Sachdev D.R., Porile N.T., Yaffe L. (1967). Reactions of ^88^Sr with protons of energies 7–85 MeV. Can. J. Chem..

[13] Levkovskii V.N. (1991). Activation Cross Sections for Nuclides of Average Masses (A = 40–100) by Protons and Alpha-Particles with Average Energies (E = 10–50 MeV).

[14] Kantelo M.V., Hogan J.J. (1976). Charged-particle emission in reaction of ^90^Zr with 10–86 MeV protons. Phys. Rev..

[15] Rösch F., Qaim S.M., Stöcklin G. (1993). Nuclear data relevant to the production of the positron emitting radioisotope ^86^Y via the ^86^Sr(p,n)- and ^nat^Rb(^3^He,xn)-processes. Radiochim. Acta.

[16] Rösch F., Qaim S.M., Stöcklin G. (1993). Production of the positron emitting radioisotope ^86^Y for nuclear medical application. Appl. Radiat. Isot..

[17] Uddin M.S., Khandaker M.U., Kim K.S., Lee Y.S., Kim G.N. (2008). Excitation functions of the proton-induced nuclear reactions on natural zirconium. Nucl. Instrum. Meth. Phys. Res..

[18] Khandaker M.U., Kim K., Lee M.W., Kim K.S., Kim G.N., Otuka N. (2009). Experimental determination of proton-induced cross sections on natural zirconium. Appl. Radiat. Isot..

[19] Szelecsényi F., Steyn G.F., Kovács Z., Vermeulen C., Nagatsu K., Zhang M.R., Suzuki K. (2015). Exciation functions of ^nat^Zr+p nuclear processes up to 70 MeV: New measurements and compilation. Nucl. Inst. Meth. Phys. Res..

[20] Tárkányi F., Ditrói F., Takács S., Hermanne A., Al-Abyad M., Yamazaki H., Baba M., Mohammadi M.A. (2015). New activation cross section data on longer lived radionuclei produced in proton induced nuclear reactions on zirconium. Appl. Radiat. Isot..

[21] Zaneb H., Hussain M., Amjed N., Qaim S.M. (2015). Nuclear model analysis of excitation functions of proton induced reactions on ^86^Sr and ^nat^Zr: Evaluation of production routes of ^86^Y. Appl. Radiat. Isot..

[22] Montgomery D.M., Porile N.T. (1969). Reactions of ^116^Cd with intermediate energy ^3^He and ^4^He ions. Nucl. Phys..

[23] Alfassi Z.B., Weinreich R. (1980). The production of positron emitters ^75^Br and ^76^Br: Excitation functions and yields for ^3^He and α-particle induced reactions on arsenic. Radiochim. Acta.

[24] Scholten B., Lambrecht R.M., Cogneau M., Vera Ruiz H., Qaim S.M. (1999). Excitation functions for the cyclotron production of ^99m^Tc and ^99^Mo. Appl. Radiat. Isot..

[25] Uddin M.S., Sudár S., Spahn I., Shariff M.A., Qaim S.M. (2016). Excitation function of the ^60^Ni(p,γ)^61^Cu reaction from threshold to 16 MeV. Phys. Rev..

[26] Finn R.D., McDevitt M., Ma D., Jurcic J., Scheinberg D., Larson S., Shoner S., Link J., Krohn K., Schlyer D., Duggan J.L., Morgan I.L. (1999). Low energy cyclotron production and separation of yttrium-86 for evaluation of monoclonal antibody pharmacokinetics and dosimetry. Applications of Accelerators in Research and Industry, Proceedings of the 15th International Conference, Woodbury, NY.

[27] Reischl G., Rösch F., Machulla H.-J. (2002). Electrochemical separation and purification of yttrium-86. Radiochim. Acta.

[28] Kettern K., Linse K.-H., Spellerberg S., Coenen H.H., Qaim S.M. (2002). Radiochemical studies relevant to the production of ^86^Y and ^88^Y at a small-sized cyclotron. Radiochim. Acta.

[29] Garmestani K., Milenic D.E., Plascjak P.S., Brechbiel W.W. (2002). A new and convenient method for purification of ^86^Y using a Sr(II) selective resin and comparison of biodistribution of ^86^Y and ^111^In labelled Herceptin^TM^. Nucl. Med. Biol..

[30] Park L.S., Szajek L.P., Wong K.J., Plascjak P.S., Garmestani K., Googins S., Eckelman W.C., Carrasquillo J.A., Paik C.H. (2004). Semi-automated ^86^Y purification using a three column system. Nucl. Med. Biol..

[31] Yoo J., Tang L., Perkins T.A., Rowland D.J., Laforest R., Lewis J.S., Welch M.J. (2005). Preparation of high specific activity ^86^Y using a small biomedical cyclotron. Nucl. Med. Biol..

[32] Avila-Rodriguez M.A., Nye J.A., Nickles R.J. (2008). Production and separation of non-carrier-added ^86^Y from enriched ^86^Sr targets. Appl. Radiat. Isot..

[33] Lukic D., Tamburella C., Buchegger F., Beyer G.-J., Comor J.J., Seimbille Y. (2009). High efficient production and purification of ^86^Y based on electrochemical separation. Appl. Radiat. Isot..

[34] Kandil S.A., Scholten B., Hassan K.F., Hanafi H.A., Qaim S.M. (2009). A comparative study on the separation of radioyttrium from Sr- and Rb-targets via ion-exchange and solvent extraction techniques, with special reference to the production of no-carrier-added ^86^Y, ^87^Y and ^88^Y using a cyclotron. J. Radioanal. Nucl. Chem..

[35] Sadeghi M., Aboudzadeh M., Zali A., Boulourinovin F. (2009). Radiochemical studies relevant to ^86^Y production via ^86^Sr(p,n)^86^Y for PET imaging. Appl. Radiat. Isot..

[36] Sadeghi M., Aboudzadeh M., Zali A., Zeinali B. (2009). ^86^Y-production via ^86^Sr(p,n) for PET imaging at a cyclotron. Appl. Radiat. Isot..

[37] Michael H., Rosezin H., Apelt H., Blessing G., Knieper J., Qaim S.M. (1981). Some technical improvements in the production of ^123^I via the ^124^Te(p,2n)^123^I reaction at a compact cyclotron. Int. J. Appl. Radiat. Isot..

[38] Spellerberg S., Scholten B., Spahn I., Bolten W., Holzgreve M., Coenen H.H., Qaim S.M. (2015). Target development for diversified irradiations at a medical cyclotron. Appl. Radiat. Isot..

[39] Vogg A.T.J., Lang R., Meier-Boeke P., Scheel W., Reske S.N., Neumaier B., Qaim S.M., Coenen H.H. (2004). Cyclotron production of radionuclides in aqueous targets matrices as alternative to solid targets. Production of ^86^Y as example. Advances in Nuclear and Radiochemistry Based on NRC-6.

[40] Oehlke E., Hoehr C., Hou X., Hanemaayer V., Zeisler S., Adam M.J., Ruth T.J., Celler A., Buckley K., Benard F. (2015). Production of ^86^Y and other radiometals for research purposes using a solution target system. Nucl. Med. Biol..

[41] Medvedev D.G., Mausner L.F., Srivastava S.C. (2011). Irradiation of strontium chloride targets at proton energies above 35 MeV to produce PET radioisotope Y-86. Radiochim. Acta.

[42] Sadeghi M., Zali A., Avila M. (2010). A novel method for radiochemical separation of radioyttrium from Sr targets using precipitation technique. Radiochim. Acta.

[43] Herzog H., Tellmann L., Scholten B., Coenen H.H., Qaim S.M. (2008). PET imaging problems with the non-standard positron emitters yttrium-86 and iodine-124. Q. J. Nucl. Med. Mol. Imaging.

[44] Lubberink M., Herzog H. (2011). Quantitative imaging of ^124^I and ^86^Y with PET. Eur. J. Nucl. Med. Mol. Imaging.

[45] Herzog H., Rösch F., Stöcklin G., Lueders C., Qaim S.M., Feinendegen L.E. (1993). Measurement of pharmacokinetics of yttrium-86 radiopharmaceuticals with PET and radiation dose calculation of analogous yttrium-90 radiotherapeutics. J. Nucl. Med..

[46] Rösch F., Herzog H., Plag C., Neumaier B., Braun U., Müller-Gärtner H.W., Stöcklin G. (1996). Radiation doses of yttrium-90 citrate and yttrium-90 EDTMP as determined via analogous yttrium-86 complexes and positron emission tomography. Eur. J. Nucl. Med..

[47] Rösch F., Herzog H., Stolz B., Brockmann J., Köhle M., Mühlensiepen H., Marbach P., Müller-Gärtner H.W. (1999). Uptake kinetics of the somatostatin receptor ligand [^86^Y]DOTA-DPhe^1^-Tyr^3^-octreotide ([^86^Y]SMT487) using positron emission tomography in non-human primates and calculation of radiation doses of the ^90^Y-labelled analogue. Eur. J. Nucl. Med..

[48] Pentlow K.S., Finn R.D., Larson S.M., Erdi Y.E., Beattie B.J., Humm J.L. (2000). Quantitative imaging of yttrium-86 with PET. The occurrence and correction of anomalous apparent activity in high density regions. Clin. Positron Imaging.

[49] Walrand S., Jamar F., Mathieu I., De Camps J., Lonneux M., Sibomana M., Labar D., Michel C., Pauwels S. (2003). Quantitation in PET using isotopes emitting prompt single gammas: Application to yttrium-86. Eur. J. Nucl. Med. Mol. Imaging.

[50] Kull T., Ruckgaber J., Weller R., Reske S., Glatting G. (2004). Quantitative imaging of yttrium-86 PET with the ECAT EXACT HR+ in 2D mode. Cancer Biother. Radiopharm..

[51] Buchholz H., Herzog H., Förster G., Reber H., Nickel O., Rösch F., Bartenstein P. (2003). PET imaging with yttrium-86: Comparison of phantom measurements acquired with different PET scanners before and after applying background. Eur. J. Nucl. Med. Mol. Imaging.

[52] IEC IS (1998). Radionuclide imaging devices characteristics and test conditions Part 1: Positron emission tomographs. Int. Electrotech. Comm..

[53] Walrand S., Flux G.D., Konijnenberg M.W., Valkema R., Krenning E.P., Lhommel R., Pauwels S., Jamar F. (2011). Dosimetry of yttrium-labelled radiopharmaceuticals for internal therapy: ^86^Y or ^90^Y imaging?. Eur. J. Nucl. Med. Mol. Imaging.

[54] Beattie B.J., Finn R.D., Rowland D.J., Pentlow K.S. (2003). Quantitative imaging of bromine-76 and yttrium-86 with PET: A method for the removal of spurious activity introduced by cascade gamma rays. Med. Phys..

[55] Ramsey N.W. (1973). Retention of ^90^Y in patients with rheumatoid arthritis. Ann. Rheum. Dis..

[56] Donaldson S.S., Chassagne D., Sancho-Garnier H., Beyer H.P. (1979). Hemangiomas of infancy: Results of ^90^Y interstitial therapy: A retrospective study. Int. J. Radiat. Oncol. Biol. Phys..

[57] Washburn L.C., Hwa Sun T.T., Crook J.E., Byrd B.L., Carlton J.E., Hung Y.W., Steplewski Z.S. (1986). ^90^Y-labeled monoclonal antibodies for cancer therapy. Int. J. Radiat. Appl. Instrum. B.

[58] ICRP Publication (1983). Radionuclide Transformations—Energy and Intensity of Emissions.

[59] Ford K. (1955). Predicted 0^+^ level in ^90^Zr. Phys. Rev..

[60] Johnson O.E., Johnson R.G., Langer L.M. (1955). Evidence for a 0^+^ first excited state in ^90^Zr. Phys. Rev..

[61] Greenberg J., Deutsch M. (1956). Positrons from the decay of ^32^P and ^90^Y. Phys. Rev..

[62] Selwyn R.G., Nickles R.J., Thomadsen B.R., DeWerd L.A., Micka J.A. (2007). A new internal pair production branching ratio of ^90^Y: The development of a non-destructive assay of ^90^Y and ^90^Sr. Appl. Radiat. Isot..

[63] Nickles R.J., Roberts A.D., Nye J.A., Converse A.K., Barnhart T.E., Avila-Rodriguez M.A., Sudaresan R., Dick D.W., Hammas R.J., Thomadsen B.R. (2004). Assaying and PET imaging of yttrium-90: 1>>34 ppm>0. IEEE Nucl. Sci. Symp. Conf. Rec..

[64] Lhommel R., Goffette P., Van den Eynde M., Jamar F., Pauwels S., Bilbao J.I., Walrand S. (2009). Yttrium-90 TOF PET scan demonstrates high resolution biodistribution after liver SIRT. Eur. J. Nucl. Med. Mol. Imaging.

[65] Werner M.K., Brechtel K., Beyer T., Dittmann K., Pfannenberg C., Kupferschläger J. (2010). PET/CT for the assessment and quantification of ^90^Y biodistribution after selective internal radiotherapy (SIRT) of liver metastases. Eur. J. Nucl. Med. Mol. Imaging.

[66] Lhommel R., van Elmbt L., Goffette P., Van den Eynde M., Jamar F., Pauwels S., Walrand S. (2010). Feasibility of ^90^Y TOF PET-based dosimetry in liver metastasis therapy using SIR-Spheres. Eur. J. Nucl. Med. Mol. Imaging.

[67] Fabbri C., Bartolomei M., Mattone V., Casi M., De Lauro F., Bartolini N., Gentili G., Amadori S., Agostini M., Sarti G. (2015). ^90^Y-PET/CT imaging quantification for dosimetry in peptide receptor radionuclide therapy: Analysis and corrections of the impairing factors. Cancer Biother. Radiopharm..

[68] Bolch W.E., Eckerman K.F., Sgouros G., Thomas S.R. (2009). MIRD Pamphlet No. 21: A generalized schema for radiopharmaceutical dosimetry—Standardization of nomenclature. J. Nucl. Med..

[69] ICRP (2007). The 2007 Recommendations of the International Commission on Radiological Protection. Ann. ICRP.

[70] Snyder W.S., Ford M.R., Warner O.G., Watson S.B. (1975). “S,” Absorbed Dose Per Unit Cumulated Activity for Selected Radionuclides and Organs.

[71] Otte A., Mueller-Brand J., Dellas S., Nitzsche E.U., Herrmann R., Maecke H.R. (1998). Yttrium-90-labelled somatostatin analogue for cancer treatment. Lancet.

[72] Förster G.J., Engelbach M.J., Brockmann J.J., Reber H.J., Buchholz H.G., Mäcke H.R., Rösch F., Herzog H.R., Bartenstein P.R. (2001). Preliminary data on biodistribution and dosimetry for therapy planning of somatostatin receptor positive tumours: Comparison of ^86^Y-DOTATOC and ^111^In-DTPA-octreotide. Eur. J. Nucl. Med..

[73] Helisch A., Förster G.J., Reber H., Buchholz H.G., Arnold R., Göke B., Weber M.M., Wiedenmann B., Pauwels S., Haus U. (2004). Pre-therapeutic dosimetry and biodistribution of ^86^Y-DOTA-Phe^1^-Tyr^3^-octreotide versus ^111^In-pentetreotide in patients with advanced neuroendocrine tumours. Eur. J. Nucl. Med. Mol. Imaging.

[74] Jamar F., Barone R., Mathieu I., Walrand S., Labar D., Carlier P., de Camps J., Schran H., Chen T., Smith M.C. (2003). ^86^Y-DOTA-D-Phe^1^-Tyr^3^-octreotide (SMT487)—A phase 1 clinical study: Pharmacokinetics, biodistribution and renal protective effect of different regimens of amino acid co-infusion. Eur. J. Nucl. Med. Mol. Imaging.

[75] Barone R., Walrand S., Konijnenberg M., Valkema R., Kvols L.K., Krenning E.P., Pauwels S., Jamar F. (2008). Therapy using labelled somatostatin analogues: Comparison of the absorbed doses with ^111^In-DTPA-D-Phe^1^-octreotide and yttrium-labelled DOTA-D-Phe^1^-Tyr^3^-octreotide. Nucl. Med. Commun..

[76] Stolz B., Bruns C., Albert R., Rösch F., Smith-Jones P., Raulf F., Hoyer D., Weckbecker G. (1999). Somatostatin receptor-targeted radiotherapy—Preclinical proof of concept. Octreotide: The Next Decade.

[77] Forrer F., Waldherr C., Maecke H.R., Mueller-Brand J. (2006). Targeted radionuclide therapy with ^90^Y-DOTATOC in patients with neuroendocrine tumors. Anticancer Res..

[78] Vinjamuri S., Gilbert T.M., Banks M., McKane G., Maltby P., Poston G., Weissman H., Palmer D.H., Vora J., Pritchard D.M. (2013). Peptide receptor radionuclide therapy with ^90^Y-DOTATATE/^90^Y-DOTATOC in patients with progressive metastatic neuroendocrine tumours: Assessment of response, survival and toxicity. Br. J. Cancer.

[79] Nayak T.K., Brechbiel M.W. (2011). ^86^Y based PET radiopharmaceuticals: Radiochemistry and biological applications. Med. Chem..

[80] Huang J., Cui L., Wang F., Liu Z. (2011). PET tracers based on ^86^Y. Curr. Radiopharm..

[81] Biddlecombe G.B., Rogers B.E., de Visser M., Parry J.J., de Jong M., Erion J.L., Lewis J.S. (2007). Molecular imaging of gastrin-releasing peptide receptor-positive tumors in mice using ^64^Cu and ^86^Y-DOTA-(Pro1, Tyr4)-bombesin (1–14). Bioconj. Chem..

[82] McQuade P., Miao Y., Yoo J., Quinn T.P., Welch M.J., Lewis J.S. (2005). Imaging of melanoma using ^64^Cu- and ^86^Y-DOTA-ReCCMSH(Arg11), a cyclized peptide analogue of alpha-MSH. J. Med. Chem..

[83] Wei L., Zhang X., Gallazzi F., Miao Y., Jin X., Brechbiel M.W., Xu H., Clifford T., Welch M.J., Lewis J.S. (2009). Melanoma imaging using ^111^In-, ^86^Y- and ^68^Ga-labeled CHX-A″-Re(Arg11)CCMSH. Nucl. Med. Biol..

[84] Banerjee S.R., Foss C.A., Pullambhatla M., Wang Y., Srinivasan S., Hobbs R.F., Baidoo K.E., Brechbiel M.W., Nimmagadda S., Mease R.C. (2015). Preclinical evaluation of ^86^Y-labeled inhibitors of prostate- specific membrane antigen for dosimetry estimates. J. Nucl. Med..

[85] Lovqvist A., Humm J.L., Sheikh A., Finn R.D., Koziorowski J., Ruan S., Pentlow K.S., Jungbluth A., Welt S., Lee F.T. (2001). PET imaging of ^86^Y-labeled anti-lewis Y monoclonal antibodies in a nude mouse model: Comparison between ^86^Y and ^111^In radiolabels. J. Nucl. Med..

[86] Nayak T.K., Regino C.A., Wong K.J., Milenic D.E., Garmestani K., Baidoo K.E., Szajek L.P., Brechbiel M.W. (2010). PET imaging of HER1-expressing xenografts in mice with ^86^Y-CHX-A″-DTPA-cetuximab. Eur. J. Nucl. Med. Mol. Imaging.

[87] Nayak T.K., Garmestani K., Milenic D.E., Baidoo K.E., Brechbiel M.W. (2011). HER1-targeted ^86^Y-panitumumab possesses superior targeting characteristics than ^86^Y-cetuximab for PET imaging of human malignant mesothelioma tumors xenografts. PLoS ONE.

[88] Nayak T.K., Garmestani K., Baidoo K.E., Milenic D.E., Brechbiel M.W. (2010). Preparation, biological evaluation, and pharmacokinetics of the human anti-HER1 monoclonal antibody panitumumab labeled with ^86^Y for quantitative PET of carcinoma. J. Nucl. Med..

[89] Schneider D.W., Heitner T., Alicke B., Light D.R., McLean K., Satozawa N., Perry G.K., Yoo J., Lewis J.S., Parry R. (2011). In vivo biodistribution, PET imaging, and tumor accumulation of Y-86- and In-111-antimindin/RG-1, engineered antibody fragments in LNCaP tumor-bearing nude mice. Int. J. Cancer.

[90] Wong K.J., Baidoo K.E., Nayak T.K., Garmestani K., Brechbiel M.W., Milenic D.E. (2011). In vitro and in vivo pre-clinical analysis of a F(ab′)_2_ fragment of panitumumab for molecular imaging and therapy of HER1 positive cancers. EJNMMI Res..

[91] McDevitt M.R., Chattopadhyay D., Jaggi J.S., Finn R.D., Zanzonico P.B., Rey D., Mendenhall J., Batt C.A., Njardson J.T., Scheinberg D.A. (2007). PET imaging of soluble yttrium-86-labeled carbon nanotubes in mice. PLoS ONE.

[92] Cheal S.M., Xu H., Guo H.-F., Lee S.-G., Punzalan B., Chalasani S., Fung E.K., Jungbluth A., Zanzonico P.B., Carrasquillo J.A. (2016). Theranostic pretargeted radioimmunotherapy of colorectal cancer xenografts in mice using picomolar affinity ^86^Y-or ^177^Lu-DOTA-Bn binding scFv C825/GPA33 IgG bispecific immunoconjugates. Eur. J. Nucl. Med. Mol. Imaging.

[93] Palm S., Enmon R.M., Matei C., Kolbert K.S., Xu S., Zanzonico P.B., Finn R.L., Koutcher J.A., Larson S.M., Sgouros G. (2003). Pharmacokinetics and biodistribution of ^86^Y-trastuzumab for ^90^Y dosimetry in an ovarian carcinoma model: Correlative MicroPET and MRI. J. Nucl. Med..

[94] Baum R.B., Rösch F. (2013). Theranostics, Gallium-68, and Other Radionuclides: A Pathway to Personalized Diagnosis and Treatment.

[95] Rösch F., Baum R.P. (2011). Generator-based PET radiopharmaceuticals for molecular imaging of tumours: On the way to THERANOSTICS. Dalton Trans..

[96] Velikyan I. (2012). Molecular imaging and radiotherapy: Theranostics for personalized patient management. Theranostics.

[97] Fani M., Pozzo L.D., Abiraj K., Mansi R., Tamma M.L., Cescato R., Waser B., Weber W.A., Reubi J.C., Mäcke H.R. (2011). PET of Somatostatin Receptor-Positive Tumors using ^64^Cu- and ^68^Ga-somatostatin antagonists: The chelate makes the difference. J. Nucl. Med..

[98] Poschenrieder A., Schottelius M., Schwaiger M., Kessler H., Wester H.-J. (2016). The influence of diferent metal-chelate conjugates of pentixafor on the CXCR4 affinity. EJNMMI Res..

[99] Filosofov D.V., Loktionova N.S., Rösch F. (2010). A ^44^Ti/^44^Sc radionuclide generator for potential application of ^44^Sc-based PET-radiopharmaceuticals. Radiochim. Acta.

[100] Rösch F. (2012). Scandium-44: Benefits of a long-lived PET radionuclide available from the ^44^Ti/^44^Sc generator system. Curr. Radiopharm..

[101] Müller C., Bunka M., Haller S., Köster U., Groehn V., Bernhardt P., van der Meulen N., Türler A., Schibli R. (2014). Promising prospects for ^44^Sc-/^47^Sc-based theragnostics: Application of ^47^Sc for radionuclide tumor therapy in mice. J. Nucl. Med..

